# Construction and equivalence for generalized boolean functions

**DOI:** 10.1007/s12095-025-00805-7

**Published:** 2025-05-22

**Authors:** Ayça Çeşmelioğlu, Wilfried Meidl

**Affiliations:** 1 Özyeğin University, Nişantepe Mah. Orman Sk., 34794 Çekmeköy, Istanbul, Turkey; 2 Institut für Mathematik, Alpen-Adria-Universität Klagenfurt, Klagenfurt, Austria

**Keywords:** EA-equivalence, CCZ-equivalence, Generalized Boolean function, Gbent function, Bent function, 06E30, 20K30, 94D10

## Abstract

Recently in Çeşmelioğlu, Meidl (*Adv. Math. Commun.,* *18*, 2024), the study of EA-equivalence and CCZ-equivalence for functions from ${\mathbb {V}}_n^{(p)}$ to the cyclic group ${\mathbb {Z}}_{p^k}$ has been initiated, where ${\mathbb {V}}_n^{(p)}$ denotes an *n*-dimensional vector space over ${\mathbb {F}}_p$. Amongst others it has been shown that there exist functions from ${\mathbb {V}}_n^{(2)}$ to ${\mathbb {Z}}_4$ which are CCZ-equivalent but not EA-equivalent. We extend these results to larger classes of functions from ${\mathbb {V}}_n^{(p)}$ to ${\mathbb {Z}}_{p^k}$. We then discuss constructions of generalized bent functions from ${\mathbb {V}}_n^{(p)}$ to ${\mathbb {Z}}_{p^k}$, *p* odd or $p=2$ and *n* is even, which correspond to large affine spaces of bent functions. In particular we employ versions of the direct sum, the semi-direct sum and of a recent secondary bent function construction in Wang et. al., (*IEEE Trans. Inform. Theory* *69*, 2023), to generate large affine spaces of bent functions. Finally we present a solution for constructing generalized bent functions from ${\mathbb {V}}_n^{(2)}$ to ${\mathbb {Z}}_{2^k}$, *n* odd, from arbitrary generalized bent functions from ${\mathbb {V}}_{n-1}^{(2)}$ to ${\mathbb {Z}}_{2^{k-1}}$.

## Introduction

Let ${\mathbb {V}}_n^{(p)}$ denote a vector space of dimension *n* over the prime field ${\mathbb {F}}_p$, and let $\langle , \rangle _n$ denote an inner product of ${\mathbb {V}}_n^{(p)}$. Standard representations of ${\mathbb {V}}_n^{(p)}$ are the vector space ${\mathbb {F}}_p^n$ of *n*-tuples over ${\mathbb {F}}_p$, and ${\mathbb {F}}_{p^n}$, the finite field with $p^n$ elements. As inner product for ${\mathbb {F}}_p^n$ we may use the conventional dot product $u\cdot v$, the standard inner product for ${\mathbb {F}}_{p^n}$ is $\langle u,v \rangle _n = \textrm{Tr}^n_1(uv)$, where $\textrm{Tr}^n_k$ denotes the trace function from ${\mathbb {F}}_{p^n}$ to ${\mathbb {F}}_{p^k}$. It is often convenient to use mixed forms, like ${\mathbb {V}}_n^{(p)} = {\mathbb {F}}_{p^m}\times {\mathbb {F}}_{p^k}$, in which case we may take $\langle (u,v), (x,y)\rangle = \textrm{Tr}^m_1(ux) + \textrm{Tr}^k_1(vy)$ as inner product.

For a function *f* from ${\mathbb {V}}_n^{(p)}$ to ${\mathbb {V}}_m^{(p)}$, the *Walsh transform* of *f* at $(a,b)\in {\mathbb {V}}_m^{(p)}\times {\mathbb {V}}_n^{(p)}$ is the complex valued function

$$\begin{aligned} \mathcal {W}_f(a,b) = \sum _{x\in {\mathbb {V}}_n^{(p)}}\zeta _p^{\langle a,f(x)\rangle _m - \langle b,x\rangle _n},\quad \zeta _p = e^{2\pi i/p}. \end{aligned}$$

If $m=1$, then the Walsh transform of *f* is defined as

$$\begin{aligned} \mathcal {W}_f(b) = \sum _{x\in {\mathbb {V}}_n^{(p)}}\zeta _p^{f(x) - \langle b,x\rangle _n}. \end{aligned}$$

The function $f:{\mathbb {V}}_n^{(p)}\rightarrow {\mathbb {V}}_m^{(p)}$ is called a *bent function* if $\mathcal {W}_f(a,b)$ has absolute value $p^{n/2}$ for all nonzero $a\in {\mathbb {V}}_m^{(p)}$ and $b\in {\mathbb {V}}_n^{(p)}$. Note that $|\mathcal {W}_f(1,b)| = p^{n/2}$ implies that $|\mathcal {W}_f(a,b)| = p^{n/2}$ for all $a\in {\mathbb {F}}_p^*$, hence for $m=1$, the bent condition reduces to $|\mathcal {W}_f(b)| = p^{n/2}$ for all $b\in {\mathbb {V}}_n^{(p)}$. For $m > 1$ the term *vectorial bent function* is common, when $m=1$, then *f* is called a *p-ary bent function* respectively a *Boolean bent function* when $p=2$.

For a Boolean bent function $f:{\mathbb {V}}_n^{(2)}\rightarrow {\mathbb {F}}_2$, which only exists if *n* is even, we have $\mathcal {W}_f(b) = 2^{n/2}(-1)^{f^*(b)}$ for a Boolean function $f^*$, called the *dual* of *f*.

Bent functions from ${\mathbb {V}}_n^{(p)}$ to ${\mathbb {F}}_p$, *p* odd, exist for even and for odd *n*. For a *p*-ary bent function $f:{\mathbb {V}}_n^{(p)}\rightarrow {\mathbb {F}}_p$ we have, see [20],

1 $$\begin{aligned} \mathcal {W}_f(b) = \left\{ \begin{array}{ll} \pm \zeta _p^{f^*(b)}p^{n/2} &  p^n \equiv 1\bmod 4, \\ \pm i\zeta _p^{f^*(b)}p^{n/2} &  p^n \equiv 3\bmod 4, \end{array}\right. \end{aligned}$$

where $f^*$ is a function from ${\mathbb {V}}_n^{(p)}$ to ${\mathbb {F}}_p$, which again is called the dual of *f*.

A bent function $f:{\mathbb {V}}_n^{(p)}\rightarrow {\mathbb {F}}_p$ is called *weakly regular*, if for all $b \in {\mathbb {V}}_n^{(p)}$ we have $\mathcal {W}_f(b) =\epsilon \ \zeta _p^{f^*(b)}p^{n/2}$ for some $\epsilon \in \{\pm 1,\pm i\}$, cf. Equation (1). If $\epsilon = 1$ we call *f* *regular*, which trivially applies if $p=2$. If (the sign of) $\epsilon$ changes with $b\in {\mathbb {V}}_n^{(p)}$, then *f* is called *non-weakly regular* bent.

Two functions $f,g:{\mathbb {V}}_n^{(p)}\rightarrow {\mathbb {V}}_m^{(p)}$ are called extended affine equivalent if

2 $$\begin{aligned} g(x) = B(f(A(x)+a)) + C(x) + c \end{aligned}$$

for some linear operators *A* on ${\mathbb {V}}_n^{(p)}$, *B* on ${\mathbb {V}}_m^{(p)}$, a linear map $C:{\mathbb {V}}_n^{(p)}\rightarrow {\mathbb {V}}_m^{(p)}$, and $a\in {\mathbb {V}}_n^{(p)}$, $c\in {\mathbb {V}}_m^{(p)}$. The functions *f*, *g* are called *CCZ-equivalent (Carlet, Charpin, Zinoviev equivalent)*, if their graphs as subsets of ${\mathbb {V}}_n^{(p)}\times {\mathbb {V}}_m^{(p)}$ are affine equivalent, cf. [5, 8].

In the last decades, a lot of research was performed on functions between elementary abelian groups ${\mathbb {V}}_n^{(p)}$ and ${\mathbb {V}}_m^{(p)}$, in particular also on bent functions. We refer to [7, 9, 20] and the references therein.

Recently one can observe a lot of interest in functions from an elementary abelian group ${\mathbb {V}}_n^{(p)}$ into the cyclic group ${\mathbb {Z}}_{p^k}$, which are often referred to as *generalized p-ary functions* or when $p=2$ as *generalized Boolean functions*. The version of the Walsh transform for a function $f:{\mathbb {V}}_n^{(p)}\rightarrow {\mathbb {Z}}_{p^k}$, called *generalized Walsh transform*, is of the form

$$\begin{aligned} \mathcal {H}_f(a,b) = \sum _{x\in {\mathbb {V}}_n^{(p)}}\zeta _{p^k}^{af(x)}\zeta _p^{\langle b,x\rangle _n},\,\zeta _q = e^{2\pi i/q}, \end{aligned}$$

$$a\in {\mathbb {Z}}_{p^k}$$, $$b\in {\mathbb {V}}_n^{(p)}$$.

The function $f:{\mathbb {V}}_n^{(p)}\rightarrow {\mathbb {Z}}_{p^k}$ is bent (also called ${\mathbb {Z}}_{p^k}$*-bent*, see [3, 21]) if $|\mathcal {H}_f(a,b)| = p^{n/2}$ for all nonzero $a\in {\mathbb {Z}}_{p^k}$ and $b\in {\mathbb {V}}_n^{(p)}$. A quite intensively studied class of functions is the class of *generalized bent (gbent) functions*, i.e., the class of functions *f* from ${\mathbb {V}}_n^{(p)}$ to ${\mathbb {Z}}_{p^k}$, which satisfy the (weaker) condition that $|\mathcal {H}_f(1,b)| = p^{n/2}$ for all $b\in {\mathbb {V}}_n^{(p)}$. By a fundamental result in [23], a gbent function from ${\mathbb {V}}_n^{(p)}$ to ${\mathbb {Z}}_{p^k}$ can be seen as a bent function *a*(*x*) from ${\mathbb {V}}_n^{(p)}$ to ${\mathbb {F}}_p$ with a corresponding partition $\mathcal {P}$ of ${\mathbb {V}}_n^{(p)}$, such that for every function $C:{\mathbb {V}}_n^{(p)}\rightarrow {\mathbb {F}}_p$, which is constant on the sets of the partition $\mathcal {P}$, the function $a(x) + C(x)$ is a bent function. The set of these bent functions forms then an affine space of bent functions.

Research on generalized Boolean functions was initiated in connection with quaternary codes and sequence sets. In particular, quaternary generalized bent functions are investigated in connection with quaternary constant-amplitude codes for code division multiple access (CDMA) systems. We refer to [16, 25, 26]. Due to the close relation to bent functions, more precisely to affine bent function spaces, generalized bent functions can be associated with objects from combinatorics, like several kinds of difference sets. The subclass of ${\mathbb {Z}}_{p^k}$-bent functions itself produces relative difference sets in ${\mathbb {V}}_n^{(p)}\times {\mathbb {Z}}_{p^k}$. When *n* is odd and $p=2$, generalized bent functions correspond to semi-bent function spaces. Large classes of Boolean and *p*-ary plateaued functions can also be obtained from plateaued generalized *p*-ary functions. Since semi-bent, and more generally plateaued functions can be balanced, they may also be interesting for cryptographic applications.

Equivalence is a crucial concept for the classification and the construction of new classes of functions between elementary abelian groups, see for instance [4–6, 8]. Investigation of equivalence for functions from ${\mathbb {V}}_n^{(p)}$ to ${\mathbb {Z}}_{p^k}$ has been initiated only recently in [10]. In particular it has been shown that, at least for $p=2$, CCZ-equivalence is more general than EA-equivalence.

In this article we extend the results in [10] on equivalence for generalized *p*-ary functions. We then investigate secondary constructions for gbent functions, seen as bent functions with a corresponding partition. In this way, we construct new (large) affine spaces of bent functions from given affine spaces of bent functions. Finally, we construct generalized bent functions from ${\mathbb {V}}_n^{(2)}$ to ${\mathbb {Z}}_{2^k}$, for odd integers *n*, from arbitrary generalized bent functions from ${\mathbb {V}}_{n-1}^{(2)}$ to ${\mathbb {Z}}_{2^{k-1}}$.

## Preliminary results

Let *f* be a function from ${\mathbb {V}}_n^{(p)}$ to ${\mathbb {Z}}_{p^k}$. Then *f* can be represented as

3 $$\begin{aligned} f(x) = a_0(x) + a_1(x)p + \cdots + a_{k-1}(x)p^{k-1} \end{aligned}$$

for uniquely determined Boolean respectively *p*-ary functions $a_i$, $0\le i\le k-1$. Clearly, the addition in (3) is the addition in ${\mathbb {Z}}_{p^k}$. As it is easily seen from the context if “$+$" refers to the addition in ${\mathbb {F}}_p$ or to the addition in ${\mathbb {Z}}_{p^k}$, we are using the same notation in both situations.

Many results on generalized *p*-ary functions *f* are obtained by connecting properties of *f* with properties of the *p*-ary functions $a_i$ in (3).

In this section we summarize results on equivalence for generalized *p*-ary functions obtained in [10], and we recall some results on generalized bent functions.

### Results on equivalence

Let $(A,+_A)$, $(B,+_B)$ be finite abelian groups, let $Aut_A$ and $Aut_B$ denote the automorphism group of *A* and *B* respectively, and with *Hom*(*A*, *B*) we denote the set of homomorphisms from *A* to *B*. Two functions *f*, *g* from *A* to *B* are extended affine equivalent if

$$\begin{aligned} g(x)=\phi _B(f(\phi _A(x)+_Aa))+_B\psi (x)+_Bb \end{aligned}$$

for some $\phi _A \in Aut_A, \phi _B \in Aut_B, \psi \in Hom(A,B), a\in A, b \in B$. If $\psi$ is the zero map, then *f* and *g* are called affine equivalent, and linear equivalent if additionally $a = 0_A$, $b = 0_B$, the zero elements of the respective groups. If *A*, *B* are elementary abelian groups, then we obtain the well-studied EA-equivalence as recalled in (2).

Two functions *f* and *g* from *A* to *B* are called CCZ-equivalent if the graph of *g*, $\mathcal {G}_g = \{(x,g(x)):\;x\in A\}$ can be obtained from the graph of *f* by applying an automorphism of $A\times B$, and a shift by a constant $(a,b)\in A\times B$.

In [10], research on EA- and CCZ-equivalence for generalized *p*-ary functions is initiated. This in particular requires knowledge on the automorphism group of ${\mathbb {V}}_n^{(p)}\times {\mathbb {Z}}_{p^k}$. We recall the corresponding result from [10] in the following proposition, where we use the representation of ${\mathbb {V}}_n^{(p)}$ via the finite field ${\mathbb {F}}_{p^n}$.

#### Proposition 1

(i): The elements of the automorphism group $Aut_{{\mathbb {F}}_{p^n}\times {\mathbb {Z}}_{p^k}}$ of ${\mathbb {F}}_{p^n}\times {\mathbb {Z}}_{p^k}$ are given by $\varphi (\mathcal {A},z,c,\gamma )$ with
  4 $$\begin{aligned} \varphi (\mathcal {A},z,c,\gamma )(x,y) = (\mathcal {A}(x) + \alpha _z(y), \beta _c(x) + \gamma y), \end{aligned}$$
  where $\mathcal {A}$ is a linearized permutation of ${\mathbb {F}}_{p^n}$, $\gamma \in {\mathbb {Z}}_{p^k}^*$, the group of the invertible elements in ${\mathbb {Z}}_{p^k}$, and for $z, c\in {\mathbb {F}}_{p^n}$, the homomorphisms $\alpha _z:{\mathbb {Z}}_{p^k}\rightarrow {\mathbb {F}}_{p^n}$ and $\beta _c:{\mathbb {F}}_{p^n}\rightarrow {\mathbb {Z}}_{p^k}$ are given by
  $$\begin{aligned} \alpha _z(y)&= z (y\bmod p), \\ \beta _c(x)&= p^{k-1}\textrm{Tr}^n_1(cx). \end{aligned}$$
(ii): Two functions *f*, *g* from ${\mathbb {F}}_{p^n}$ to ${\mathbb {Z}}_{p^k}$ are CCZ-equivalent if
  $$\begin{aligned} \{(x,g(x))\;:\;x\in {\mathbb {F}}_{p^n}\} = \{\varphi (\mathcal {A},z,c,\gamma )(x,f(x)) + (u,v)\;:\; x\in {\mathbb {F}}_{p^n}\}, \end{aligned}$$
  for some automorphism $\varphi (\mathcal {A},z,c,\gamma )$ of ${\mathbb {F}}_{p^n}\times {\mathbb {Z}}_{p^k}$ and $(u,v)\in {\mathbb {F}}_{p^n}\times {\mathbb {Z}}_{p^k}$. If $z=0$, then *f* and *g* are EA-equivalent.

Similar as for CCZ-equivalence for functions between elementary abelian groups, several interesting properties are invariant under CCZ-equivalence for generalized *p*-ary functions. For instance the property of being gbent (${\mathbb {Z}}_{p^k}$-bent) is invariant under CCZ-equivalence.

As for the case of functions between elementary abelian groups, applying a given automorphism of $Aut_{{\mathbb {F}}_{p^n}\times {\mathbb {Z}}_{p^k}}$ to the graph of a given function $f:{\mathbb {V}}_n^{(p)}\rightarrow {\mathbb {Z}}_{p^k}$ in general does not yield the graph of a function. Let *f* be given as in (3). As can be easily observed from (4), $\varphi (\mathcal {A},z,c,\gamma )(x,f(x))$ is the graph of a function if and only if $\mathcal {A}(x) + \alpha _z(f(x)) = \mathcal {A}(x) + za_0(x)$ is a permutation of ${\mathbb {F}}_{p^n}$. The following proposition is Theorem 3.5 and Corollary 4.5 in [10].

#### Proposition 2

Let $f:{\mathbb {F}}_{p^n}\rightarrow {\mathbb {Z}}_{p^k}$ be given as in (3), and let $\varphi (\mathcal {A},z,c,\gamma ) \in Aut_{{\mathbb {F}}_{p^n}\times {\mathbb {Z}}_{p^k}}$.

(i): Applying $\varphi (\mathcal {A},z,c,\gamma )$ to the graph of *f* yields the graph of a function if and only if the *p*-ary function $a_0$ in (3) satisfies
  5 $$\begin{aligned} a_0(x) = a_0(x+\kappa \mathcal {A}^{-1}(z)) \end{aligned}$$
  for all $x\in {\mathbb {F}}_{p^n}$ and for all $\kappa \in {\mathbb {F}}_p$.
(ii): If $\mathcal {A}(x) + za_0(x)$ is not a permutation for any linearized permutation $\mathcal {A}$ of ${\mathbb {F}}_{p^n}$ and nonzero element $z\in {\mathbb {F}}_{p^n}$, then any function *g* which is CCZ-equivalent to *f*, is EA-equivalent to *f*.

As a main result in [10] it is shown that in general, CCZ-equivalence for generalized *p*-ary functions is coarser than EA-equivalence. More precisely, we have the following theorem.

#### Theorem 1

Let $A = {\mathbb {V}}_4^{(2)}\times G$ and $B = {\mathbb {Z}}_4\times H$ for some finite abelian groups *G*, *H*. Then there exist pairs of functions $f,g: A\rightarrow B$ which are CCZ-equivalent, but not EA-equivalent. In particular, there exist pairs of functions from ${\mathbb {V}}_n^{(2)}$ to ${\mathbb {Z}}_4$ which are CCZ-equivalent, but not EA-equivalent.

We close this subsection with some more results from [10] on EA- and CCZ-equivalence for generalized *p*-ary functions, some of which were used to show Theorem 1. Let *f* be a generalized *p*-ary function given as in (3). Then we can associate with *f* the vectorial function $F:{\mathbb {F}}_{p^n}\rightarrow {\mathbb {F}}_p^k$ given as $F(x) = (a_0(x),a_1(x),\ldots ,a_{k-1}(x))$. We denote with the component functions of *f*, the component functions of the vectorial function *F*. The algebraic degree of *f* is the algebraic degree of *F*, i.e., the maximal algebraic degree among the algebraic degrees of the component functions of *F*. As shown in [10], EA-equivalence (CCZ-equivalence) of $f,g:{\mathbb {F}}_{p^n}\rightarrow {\mathbb {Z}}_{p^k}$ implies EA-equivalence (CCZ-equivalence) of the corresponding vectorial functions *F*, *G*.

#### Proposition 3

Let $f(x) = a_0(x) + a_1(x)p + \cdots + a_{k-1}(x)p^{k-1}$ be a generalized *p*-ary function.

(i): The algebraic degree of *f* is invariant under EA-equivalence (cf. [10, Corollary 4.3]), but not under CCZ-equivalence.
(ii): The number of bent components in the corresponding vectorial function $F(x) = (a_0(x),a_1(x),\ldots ,a_{k-1}(x))$ is invariant under EA-equivalence (cf. [10, Corollary 4.3]), but not under CCZ-equivalence.
(iii): If $a_0$ is an affine function, and $g:{\mathbb {F}}_{p^n} \rightarrow {\mathbb {Z}}_{p^k}$ and *f* are CCZ-equivalent, then they are EA-equivalent, cf. [10, Corollary 4.7].
(iv): If $a_0$ is a bent function, and $g:{\mathbb {F}}_{p^n} \rightarrow {\mathbb {Z}}_{p^k}$ and *f* are CCZ-equivalent, then they are EA-equivalent. In particular, two ${\mathbb {Z}}_{p^k}$-bent functions are CCZ-equivalent if and only if they are EA-equivalent, cf. [10, Theorem 4.8].

### Results on gbent functions

Generalized bent functions were originally introduced as functions *f* from ${\mathbb {V}}_n^{(p)}$ to ${\mathbb {Z}}_{p^k}$ for which $\mathcal {H}_f(1,u)$ has absolute value $p^{n/2}$ for all $u\in {\mathbb {V}}_n^{(p)}$. Trivial (but not very interesting) examples satisfying this condition are $f(x) = p^{k-1}g(x)$ for a bent function $g:{\mathbb {V}}_n^{(p)}\rightarrow {\mathbb {F}}_p$. In several articles, gbent functions *f* are characterized in terms of the *p*-ary functions $a_j$ in the representation given in (3). When *p* is odd, or $p=2$ and *n* is even, then a necessary condition for *f* to be a gbent function is that

6 $$\begin{aligned} A_f = a_{k-1}(x) + \langle a_0(x), \ldots , a_{k-2}(x)\rangle \end{aligned}$$

is an affine space of bent functions. Clearly, the dimension of this affine bent function space is at most $k-1$. Technical sufficient conditions on such an affine space to represent a generalized bent function are given e.g. in [18, 27] in terms of the values of the Walsh transforms of the *p*-ary bent functions $a_{k-1}(x) + \sum _{i=0}^{k-2}c_ia_i(x)$, $c_i\in {\mathbb {F}}_p$.

The characterization of generalized bent functions via an affine space $A_f$ in (6) turned out to be very useful. Several interesting results have been obtained using this description.

A breakthrough for a comprehensive understanding of generalized bent functions is achieved in Mesnager et al. [23]. Let $f:{\mathbb {V}}_n^{(p)}\rightarrow {\mathbb {Z}}_{p^k}$ be given as

$$\begin{aligned} f(x) = a_0(x) + a_1(x)p + \cdots + a_{k-1}(x)p^{k-1}, \end{aligned}$$

then we can assign to *f* a partition $\mathcal {P} = \{A(d):\,0\le d\le p^{k-1}-1\}$ of ${\mathbb {V}}_n^{(p)}$, where

7 $$\begin{aligned} A(d) = \left\{ x\in {\mathbb {V}}_n^{(p)}\,:\,\sum _{i=0}^{k-2}a_i(x)p^i = d\right\} , \end{aligned}$$

Note that some of the sets *A*(*d*) may be empty, hence $\mathcal {P}$ is a partition of ${\mathbb {V}}_n^{(p)}$ into at most $p^{k-1}$ sets.

We then have the following theorem.

#### Theorem 2

 [23, Theorem 16] Let *p* be odd, or $p=2$ and *n* even, and let $f:{\mathbb {V}}_n^{(p)}\rightarrow {\mathbb {Z}}_{p^k}$ be given as in (3).

Then *f* is generalized bent, if and only if $a_{k-1}(x) + C(x)$ is bent for every *p*-ary respectively Boolean function *C*(*x*), which is constant on the sets *A*(*d*) in (7) of the partition $\mathcal {P}$.

By Theorem 2, when *p* is odd or $p=2$ and *n* is even, then a generalized bent function is a *p*-ary bent function $a(x) = a_{k-1}(x)$ from ${\mathbb {V}}_n^{(p)}$ to ${\mathbb {F}}_p$ together with a partition of ${\mathbb {V}}_n^{(p)}$. In accordance with [23] we say that the bent function *a* is *admissible relative to the partition* of ${\mathbb {V}}_n^{(p)}$, which we denote by $\mathcal {P}_a$, or by $\mathcal {P}_a^f$ if we want to emphasize that the partition is determined as in (7) from a function *f* given as in (3). In the light of Theorem 2 we will denote a gbent function also by $(a,\mathcal {P}_a)$ or by $(a,\mathcal {P}^f_a)$.

#### Remark 1

Let $p=2$ and *n* be even, or let *p* be odd and *n* be an arbitrary integer, and let $a:{\mathbb {V}}_n^{(p)}\rightarrow {\mathbb {F}}_p$ be a bent function admissible relative to the partition $\mathcal {P}_a$ of ${\mathbb {V}}_n^{(p)}$.

(i): Clearly, many choices for $a_0,a_1,\ldots ,a_{k-2}$ in (7), give rise to the same partition of ${\mathbb {V}}_n^{(p)}$. To represent a partition $\mathcal {P}$ of ${\mathbb {V}}_n^{(p)}$ of cardinality $|\mathcal {P}|= K$, it requires $\lceil \log _pK\rceil$ *p*-ary functions. It is meaningless to represent $\mathcal {P}$ with a larger number of $a_i$ (e.g. choosing some $a_i$ as the 0-function), but not contradictory.
(ii): The set of bent functions
  $$\begin{aligned} \{a(x) + C(x)\,:\, C\,\text{ constant } \text{ on } \text{ the } \text{ sets } \text{ of } \text{ the } \text{ partition }\,\mathcal {P}_a\} \end{aligned}$$
  can alternatively be represented as the set
  $$\begin{aligned} \{ a(x) + F(a_0(x),\ldots ,a_{k-2}(x))\,:\,F:{\mathbb {F}}_p^{k-1}\rightarrow {\mathbb {F}}_p\}. \end{aligned}$$
  This set forms an affine vector space of bent functions of dimension $|\mathcal {P}_a|$, and following Theorem 2 we can identify a generalized bent function with the induced affine space, which we denote by $\mathcal {A}(a,\mathcal {P}_a)$.
(iii): The description of a generalized bent function as the affine space $A_f$ in (6) captures only a small fraction of the whole object. As obvious, $A_f$ in (6) is a (small) subspace (of dimension at most $k-1$) of $\mathcal {A}(a_{k-1},\mathcal {P}^f_{a_{k-1}})$. Hence, a complete treatment of generalized bent functions inevitably includes the study of partitions corresponding to bent functions.

#### Remark 2

We introduced three notations for a gbent function which we will use ambiguously.

- We use the formula $f(x) = a_0(x) + a_1(x)p + \cdots + a_{k-1}(x)p^{k-1}$ of a gbent function as a function mapping from ${\mathbb {V}}_n^{(p)}$ to ${\mathbb {Z}}_{p^k}$;
- We write $(a,\mathcal {P}_a)$ to indicate a bent function $a:{\mathbb {V}}_n^{(p)}\rightarrow {\mathbb {F}}_p$, which is admissible relative to the partition $\mathcal {P}_a$ of ${\mathbb {V}}_n^{(p)}$;
- We write $\mathcal {A}(a,\mathcal {P}_a)$ if it is important to emphasize the affine space of bent functions of dimension $|\mathcal {P}_a|$ induced by $(a,\mathcal {P}_a)$.

All three notations describe the same object, with the constraint that there are various ways to represent $(a,\mathcal {P}_a)$, $\mathcal {A}(a,\mathcal {P}_a)$ with a formula for *f*(*x*). Note also that every bent function $\tilde{a} \in \mathcal {A}(a,\mathcal {P}_a)$ is admissible relative to $\mathcal {P}_a$, hence $\mathcal {A}(a,\mathcal {P}_a) = \mathcal {A}(\tilde{a},\mathcal {P}_a)$. In this connection we may refer to our Proposition 5 below.

## More results on equivalence

In [10] an example of two functions $f,g:{\mathbb {F}}_{2^4}\rightarrow {\mathbb {Z}}_4$ which are CCZ-equivalent but not EA-equivalent is constructed. Using a result in [24], it is pointed out that there exist functions from ${\mathbb {V}}_n^{(2)}$ to ${\mathbb {Z}}_4$ which are CCZ-equivalent but not EA-equivalent for arbitrary integers *n*.

In the following we extend this result. We describe a procedure to construct functions from ${\mathbb {V}}_n^{(p)}$ to ${\mathbb {Z}}_{p^k}$ which are CCZ-equivalent but not EA-equivalent for small values of *n*. With the same argument as in [10], this result can be extended to arbitrary *n*. We point to the difficulties in this procedure, and apply it for $p = 2,3,5$. The procedure is based on the following proposition.

### Proposition 4

Let $a_0:{\mathbb {V}}_n^{(p)}\rightarrow {\mathbb {F}}_p$, and a linearized permutation $\mathcal {A}$ of ${\mathbb {F}}_{p^n}$ and $z\in {\mathbb {F}}_{p^n}$ be such that $\wp (x) = \mathcal {A}(x) + za_0(x)$ is a permutation of ${\mathbb {F}}_{p^n}$. Suppose that $a_0$ has algebraic degree $\deg (a_0) \ge 2$ and let $a_i:{\mathbb {V}}_n^{(p)}\rightarrow {\mathbb {F}}_p$ be such that $\deg (a_i) \le \deg (a_0)$ for $1\le i\le k-1$. If $\deg (a_1(\wp ^{-1})) > \deg (a_0)$, then

$$\begin{aligned} f(x)&= a_0(x) + a_1(x)p + \cdots + a_{k-1}(x)p^{k-1} \quad \text{ and } \\ g(x)&= a_0(\wp ^{-1}(x)) + a_1(\wp ^{-1}(x))p + \cdots + a_{k-2}(\wp ^{-1}(x))p^{k-2} \\&+ (a_{k-1}(\wp ^{-1}(x)) + \textrm{Tr}^{n}_1(\wp ^{-1}(x)))p^{k-1} \end{aligned}$$

are CCZ-equivalent but not EA-equivalent.

### Proof

Choose the linearized permutation $\mathcal {A}$ of ${\mathbb {F}}_{p^n}$ and a fixed element $z \in {\mathbb {F}}_{p^n}$. Then the function $a_0:{\mathbb {V}}_n^{(p)}\rightarrow {\mathbb {F}}_p$ constructed as in Proposition 2 (i), yields a permutation $\wp (x) = \mathcal {A}(x) + za_0(x)$. By applying the automorphism $\varphi (\mathcal {A},z,c,\gamma ) \in Aut_{{\mathbb {F}}_{p^n}\times {\mathbb {Z}}_{p^k}}$ to the graph of *f* with these choices for $\mathcal {A}$ and *z*, and by choosing $c\in {\mathbb {F}}_{p^n}, \gamma \in {\mathbb {Z}}_{p^k}^*$ as $c=1, \gamma =1$ and $(u,v)=(0,0)$, we obtain

$$\begin{aligned} \{(x,g(x)) :\;x\in {\mathbb {F}}_{p^n}\}&= \{\varphi (\mathcal {A},z,1,1)(x,f(x)) :\;x\in {\mathbb {F}}_{p^n}\} \\&= \{(\mathcal {A}(x) + za_0(x), p^{k-1}\textrm{Tr}_1^n(x) + f(x)) :\;x\in {\mathbb {F}}_{p^n}\} \\&= \{(\wp (x), p^{k-1}\textrm{Tr}_1^n(x) + f(x)):\;x\in {\mathbb {F}}_{p^n}\}. \end{aligned}$$

Therefore

$$\begin{aligned} g(x)&= p^{k-1}\textrm{Tr}_1^n(\wp ^{-1}(x)) + f(\wp ^{-1}(x))\\&= a_0(\wp ^{-1}(x)) + a_1(\wp ^{-1}(x))p + \cdots + (a_{k-1}(\wp ^{-1}(x)) + \textrm{Tr}_1^n(\wp ^{-1}(x)))p^{k-1}. \end{aligned}$$

The functions *f* and *g* are CCZ-equivalent. If the conditions in the proposition are satisfied then the algebraic degrees of *f* and *g* are different, which implies that *f* and *g* are not EA-equivalent. $\Box$

With Proposition 4 we infer the following procedure to construct CCZ-equivalent but not EA-equivalent functions from ${\mathbb {V}}_n^{(p)}$ to ${\mathbb {Z}}_{p^k}$.

**Step 1.**: Construct the permutation $\wp (x)$. To construct $\wp (x) = \mathcal {A}(x) + za_0(x)$, we fix the automorphism (linearized permutation) $\mathcal {A}$ of ${\mathbb {V}}_n^{(p)}$ and $z\in {\mathbb {V}}_n^{(p)}$. We partition ${\mathbb {V}}_n^{(p)}$ into $p^{n-1}$ equivalence classes each of *p* elements, where the class of an element $x\in {\mathbb {V}}_n^{(p)}$ is given as
  $$\begin{aligned} E_x = \{x + \kappa \mathcal {A}^{-1}(z)\,:\,\kappa \in {\mathbb {F}}_p\}. \end{aligned}$$
  We then choose for $a_0$ a *p*-ary function which is constant on the sets $E_x$, such that $a_0$ satisfies (5). By Proposition 2, applying $\varphi (\mathcal {A},z,c,\gamma )$ to the graph of *f* yields the graph of a function, equivalently, $\wp (x) = \mathcal {A}(x) + za_0(x)$ is a permutation of ${\mathbb {V}}_n^{(p)}$. In this way, with different choices of $\mathcal {A}, z$, many permutations $\wp$ can be constructed. The difficulty in this step, and in the whole procedure, is to guarantee that $a_0$ has small or at least moderately large algebraic degree $\deg (a_0) \ge 2$, so that we can find $a_1$ satisfying the required properties in the next step. Many attempts with various choices for $\mathcal {A}$ and *z* may be needed.
**Step 2.**: Choose $a_1(x)$. We require that $\deg (a_1) \le \deg (a_0)$ and $\deg (a_1(\wp ^{-1})) > \deg (a_0)$. Constructing $a_1$ with these properties seems not easy, but it seems easy to find such $a_1$ with a random choice. Then for $i=2, \ldots , k-1$ choosing $a_i$ of algebraic degree at most $\deg (a_0)$ (e.g. $a_i$ constant), *f*, *g* given as in Proposition 4 are CCZ-equivalent, but not EA-equivalent. Applying this procedure, we derive the following examples in characteristic $p=2$, $p=3$ and $p=5$:

### Example 1

$$f,g:{\mathbb {F}}_{2^4}\rightarrow {\mathbb {Z}}_{2^3}$$

We use the representation ${\mathbb {F}}_{2^4}={\mathbb {F}}_2(w)$, where *w* is a root of the polynomial $x^4+x+1 \in {\mathbb {F}}_2[x]$.

*Step 1:* We fix $\mathcal {A}$ as the linearized permutation $\mathcal {A}(x)=x^8+wx^2$, and we choose $z = w$. Then $\mathcal {A}^{-1}(z)=\mathcal {A}^{-1}(w)=w^{14}$. The equivalence classes $E_x$ are then given by the sets
  $$\begin{aligned} E_0&=\{0,w^{14}\}, E_1=\{1,w^3\}, E_w=\{w,w^7\}, E_{w^2}=\{w^2, w^{13}\}, \\ E_{w^4}&= \{w^4,w^9\}, E_{w^5}=\{w^5, w^{12}\}, E_{w^6}=\{w^6,w^8\}, E_{w^{10}}=\{w^{10}, w^{11}\}. \end{aligned}$$
  To construct the permutation
  $$\begin{aligned} \wp (x) = \mathcal {A}(x)+wa_0(x)=x^8+ wx^2 + wa_0(x), \end{aligned}$$
  we choose the Boolean function $$a_0(x)$$ which takes the value 1 on $$E_1, E_{w^2}, E_{w^5},$$$$ E_{w^{10}}$$ and which is 0 on $$E_0, E_w, E_{w^4}, E_{w^6}$$. Then
  $$\begin{aligned} a_0(x)&= (w^{3} + 1)x^{12}+ (w^{2} + w)x^{10} + (w^{3} + w^2+1)x^{9} + (w^{3} + 1)x^{8} \\&+ (w^3 + w + 1)x^{6} + (w^2 + w + 1) x^5 + (w^3 + w + 1)x^4 + (w^3 + w^2 + w)x^3 \\&+ (w^3 + w^2 + w)x^2 + (w^3 + w^2 + 1)x. \end{aligned}$$
  Note that the algebraic degree of $a_0$ is $\deg (a_0) = 2$ and its Walsh spectrum is given as $\{-8^1, 0^{12}, 8^3\}$, where the entry, e.g., $8^3$ means that 8 appears in the Walsh spectrum 3 times.
*Step 2:* For $a_1$ we take the quadratic bent function
  $$\begin{aligned} a_1(x) = \textrm{Tr}^4_1(w^4x^3), \end{aligned}$$
  and $a_2(x) = 1$, so that we have a function $f(x) = a_0(x) + 2a_1(x) + 4a_2(x)$ that maps to ${\mathbb {Z}}_8$. We apply the automorphism $\varphi (\mathcal {A},z,c,\gamma )$ with $\gamma = 1, c = 1$, and obtain the function $g:{\mathbb {F}}_{2^4}\rightarrow {\mathbb {Z}}_{2^3}$ given as
  $$\begin{aligned} g(x)&= a_0(\wp ^{-1}(x)) + 2a_1(\wp ^{-1}(x)) + 2^2(a_2(\wp ^{-1}(x)) + \textrm{Tr}^4_1(\wp ^{-1}(x))\\&= b_0(x) + 2b_1(x) + 2^2b_2(x), \end{aligned}$$
  which is CCZ-equivalent to *f*(*x*).

We have,

$$\begin{aligned} a_0(\wp ^{-1}(x))&= (w^2 + 1)x^{12} + (w^2 + w)x^{10} + wx^9 + x^8 + (w + 1)x^6 + (w^2 + w + 1)x^5 + x^4 \\&+ w^2x^3 + x^2 + x, \\ a_1(\wp ^{-1}(x))&=(w^3 + w^2 + w + 1)x^{14} + (w^3 + w)x^{13} + (w^2 + w)x^{12} + w^3x^{11} + x^{10} \\&+ (w^2 + w + 1)x^9 + (w^3 + w^2 + w + 1)x^8 + (w^3 + w^2)x^7 + (w^2 + w + 1)x^6 \\&+ x^5 + (w^3 + w^2)x^4 + (w^2 + w)x^3 + w^3x^2 + (w^3 + w)x, \end{aligned}$$

and with $a_2(\wp ^{-1}(x)) = 1$ we have

$$\begin{aligned} a_2(\wp ^{-1}(x)) + \textrm{Tr}^4_1(\wp ^{-1}(x)) = 1 + \textrm{Tr}^4_1(\wp ^{-1}(x)), \end{aligned}$$

where

$$\begin{aligned} \textrm{Tr}^4_1(\wp ^{-1}(x))&= (w^2 + 1)x^{12} + (w^2 + w)x^{10} + wx^9 + (w^3 + 1)x^8 + (w + 1)x^6 \\&+ (w^2 + w + 1)x^5 + (w^3 + w + 1)x^4 + w^2x^3 + (w^3 + w^2 + w)x^2 \\&+ (w^3 + w^2 +1)x. \end{aligned}$$

Below we list the algebraic degree and Walsh spectrum of all components of *g*.

**Table:** 

Component	Degree	Walsh spectrum
$$b_0$$	2	$$ -8^1, 0^{12}, 8^3$$
$$b_1$$	3	$$ -8^1, -4^2, 0^6, 4^6, 8^1$$
$$b_2$$	2	$$ -8^3, 0^{12}, 8^1$$
$b_0+b_1$	3	$-4^4, 0^6, 4^4, 8^2$
$b_0+b_2$	2	$-16^1, 0^{15}$
$$b_1+b_2$$	3	$$ -8^2, -4^4, 0^6, 4^4$$
$$b_0+b_1+b_2$$	3	$$-8^1, -4^6, 0^6, 4^2, 8^1 $$

Observe that whereas *f* has algebraic degree 2 and the bent components $a_1$, $a_1+a_2$, the function *g* has algebraic degree 3 and no bent component. The CCZ-equivalent functions *f*, *g* are hence not EA-equivalent. We remark that *f*, *g* have the same Walsh spectrum (collections of the values in the Walsh spectra of the components), namely $\{16^1,-8^8,-4^{16},0^{63},4^{16},8^8\}$.

With the same choice for $\mathcal {A}$, *z* and $a_0$, we can similarly construct CCZ-equivalent but not EA-equivalent functions *f*, *g* from ${\mathbb {F}}_{2^4}$ to ${\mathbb {Z}}_{2^k}$, $k>2$, for instance choosing $a_i(x) = 1$ for $2\le i\le k-1$.

### Example 2

$$f,g:{\mathbb {F}}_{3^2}\rightarrow {\mathbb {Z}}_{3^2}$$

We consider ${\mathbb {F}}_{3^2}={\mathbb {F}}_3(w)$, where *w* is a root of the polynomial $x^2+2x+2 \in {\mathbb {F}}_3[x]$.

*Step 1:* We choose $\mathcal {A}(x) = x + wx^3$. If we choose $z=w$, then $\mathcal {A}^{-1}(z) =\mathcal {A}^{-1}(w) = w^6$, and the equivalence classes $E_x$ are then given by
  $$\begin{aligned} E_0&=\{0, w^6, 2w^6\} = \{0, w^6, w^2\}, E_1 = \{1, w^6+1, 2w^6+1\} = \{1, w^5, w^7\},\\ E_2&= \{2, w^6 + 2, 2w^6 + 2\} = \{2, w, w^3\}. \end{aligned}$$
  We choose $a_0$ such that $a_0(x) = 0$ for $x\in E_0$, and $a_0(x) = 2$ for $x\in E_1\cup E_2$, which gives $a_0(x) = 2x^6 + x^4 + 2x^2$. The Walsh spectrum of $a_0$ is given as $\{ 0^6, (-6\zeta _3 - 3)^1, (3\zeta _3 + 6)^2\}$. The permutation $\wp$ of ${\mathbb {F}}_{3^2}$ is then
  $$\begin{aligned} \wp (x) = \mathcal {A}(x) + wa_0(x)= x + wx^3 + wa_0(x). \end{aligned}$$
*Step 2.*: For $a_1$ we take the quadratic bent function $a_1(x) = \textrm{Tr}^2_1(x^2)$. Applying $\varphi (A,z,c,\gamma )$ with $\gamma = 1, c = 1$ to $f(x) = a_0(x) + 3a_1(x)$, we obtain
  $$\begin{aligned} g(x)&= a_0(\wp ^{-1}(x)) + 3(a_1(\wp ^{-1}(x))+ \textrm{Tr}^2_1(\wp ^{-1}(x)))\\&= b_0(x) + 3b_1(x), \end{aligned}$$
  which is CCZ-equivalent to *f*. We have,
  $$\begin{aligned} b_0(x)&= a_0(\wp ^{-1}(x)) = w^2x^6 + 2x^4 + w^6x^2, \end{aligned}$$
  and with
  $$\begin{aligned} a_1(\wp ^{-1}(x)) = wx^7 + x^6 + w^3x^5 + 2x^4 + w^6x^3 + x^2 + w^2x,\, \textrm{Tr}^2_1(\wp ^{-1}(x)) = w^7x^3 + w^5x \end{aligned}$$
  we have
  $$\begin{aligned} b_1(x) = a_1(\wp ^{-1}(x)) + \textrm{Tr}^2_1(\wp ^{-1}(x)) = wx^7 + x^6 + w^3x^5 + 2x^4 + x^3 + x^2 + x. \end{aligned}$$

For the algebraic degree and Walsh spectra we have

**Table:** 

Component	Degree	Walsh spectrum
$b_0$	2	$0^6, (-6\zeta _3-3)^1, (3\zeta _3+6)^2$
$2b_0$	2	$0^6, (-3\zeta _3+3)^2, (6\zeta _3+3)^1$
$b_1$	3	$0^2, 3^1, (-3\zeta _3)^2, (-3\zeta _3+1)^1, (3\zeta _3)^2, (3\zeta _3+3)^1$
$2b_1$	3	$0^2, 3^1, (-3\zeta _3-3)^2, (-3\zeta _3)^1, (3\zeta _3+3)^2, (3\zeta _3+6)^1$
$b_0+b_1$	3	$0^2, (-3)^1, (3)^2, (-3\zeta _3-3)^1, (-3\zeta _3+3)^1, (3\zeta _3+3)^2$
$b_0+2b_1$	3	$0^2, 3^2, (-6\zeta _3-3)^1, (-3\zeta _3)^1, (3\zeta _3)^1, (3\zeta _3+3)^2$
$2b_0+b_1$	3	$0^2, 3^2, (-3\zeta _3-3)^2, (-3\zeta _3)^1, (3\zeta _3+3)^1, (6\zeta _3+3)^1$
$2b_0+2b_1$	3	$0^2, (-3)^1, (3)^2, (-3\zeta _3)^{2}, (3\zeta _3)^1, (3\zeta _3+6)^1$

Again with the algebraic degree argument, *f* and *g* are not EA-equivalent.

### Example 3

$$f,g: {\mathbb {F}}_{5^2} \rightarrow {\mathbb {Z}}_{5^2}$$

Let *w* be a root of the irreducible polynomial $x^2+4x+2 \in {\mathbb {F}}_5[x]$ and ${\mathbb {F}}_{5^2}={\mathbb {F}}_5[w]$.

*Step 1.*: We choose $\mathcal {A}(x) = x^5+wx \in {\mathbb {F}}_{5^2}[x]$ and $z=w$. Then $\mathcal {A}^{-1}(w)=w^{22}$, and
  $$\begin{aligned}&E_0=\{0, w^{22}, w^{4}, w^{16}, w^{10}\}, E_1=\{1, w^{15}, w^{23}, w^{20}, w^{13}\}, E_2=\{2, w^{2}, w^{21}, w^{19}, w^{5}\}, \\&E_3=\{3, w^{17}, w^{7}, w^{9}, w^{14}\}, E_4=\{4, w, w^{8}, w^{11}, w^{3}\}. \end{aligned}$$
  We choose $a_0$ for which $a_0(x) = 0$ for $x \in E_0\cup E_1$, $a_0(x) = 1$ for $x\in E_2$, $a_0(x) = 2$ for $x\in E_3$, and $a_0(x) = 4$ for $x\in E_4$, and compute the resulting polynomial as
  $$\begin{aligned} a_0(x)&=w^{22}x^{20} + w^{14}x^{16} + w^3x^{15} + 2x^{12} + wx^{11} + w^{14}x^{10} + w^{22}x^8 + w^5x^7 \\&+ x^6 + w^7x^5 + w^{14}x^4 + w^{15}x^3 + w^{22}x^2 + w^{11}x. \end{aligned}$$
  The Walsh spectrum of $a_0$ is $\{0^{20}, (-15\zeta _5^3 - 15\zeta _5^2 - 15\zeta _5 - 5)^1, (-5\zeta _5^3 + 5)^1, (5\zeta _5^3 + 5\zeta _5^2 + 10\zeta _5 + 5)^1, (5\zeta _5^3 + 10\zeta _5^2 + 10)^1, (10\zeta _5^3 + 5\zeta _5 + 10)^1\}$. The corresponding permutation polynomial $\wp$ over ${\mathbb {F}}_{5^2}$ is hence $\wp (x) = \mathcal {A}(x) + za_0(x)$, and its inverse is
  $$\begin{aligned} \wp ^{-1}(x)&=4x^{24} + 2x^{23} + x^{22} + 3x^{21} + w^{16}x^{20} + 2x^{19} + x^{18} + 3x^{17} + w^2x^{15}\\&+ x^{14} + 3x^{13} + w^8x^{12} + w^{23}x^{11} + 3x^{10} + 3x^9 + w^7x^8 + 3x^7 + w^{23}x^6\\&+ w^9x^5 + 3x^4 + w^2x^3 + w^3x^2 + w^3x. \end{aligned}$$
*Step 2.*: We choose $a_1(x) = x$, then $1 = \deg (a_1) \le \deg (a_0) = 4$ and $8 = \deg (a_1(\wp ^{-1})) = \deg (\wp ^{-1}) > \deg (a_0) = 4$. We apply $\varphi (\mathcal {A},z,c,\gamma )$ with $c=1$, $\gamma = 1$, and obtain $g(x) = b_0(x) + 5b_1(x) = a_0(\wp ^{-1}(x)) + 5(a_1(\wp ^{-1}(x)) + \textrm{Tr}_1^2(\wp ^{-1}(x)))$ with
  $$\begin{aligned} b_0(x)&= a_0(\wp ^{-1}(x)) = 3x^{24} + 4x^{23} + 2x^{22} + x^{21} + w^{14}x^{20} + 4x^{19} + 2x^{18} + x^{17} \\&+ w^{13}x^{16} + 3x^{15} + 2x^{14} + x^{13} + x^{12} + wx^{11} + w^9x^{10} + x^9 + w^{17}x^8 \\&+ w^5x^7 + w^{13}x^5 + w^{22}x^4 + 3x^3 + w^{21}x^2 + w^{17}x \quad \text{ and } \\ b_1(x)&= a_1(\wp ^{-1}(x)) + \textrm{Tr}_1^2(\wp ^{-1}(x)) = \wp ^{-1}(x) + \textrm{Tr}_1^2(\wp ^{-1}(x)) . \end{aligned}$$
  Observing that *f* has algebraic degree 4 and *g* has algebraic degree 8, we confirm that *f* and *g* are not EA-equivalent.

As our examples show, for generalized *p*-ary functions, both in even and odd characteristic, in general CCZ-equivalence is coarser then EA-equivalence. In the form of a theorem, we can state the following:

### Theorem 3

Let $n\ge 2$ when $p=3,5$, and $n\ge 4$ when $p=2$, and let $k\ge 2$. Then there exist generalized *p*-ary functions *f*, *g* from ${\mathbb {V}}_n^{(p)}$ to ${\mathbb {Z}}_{p^k}$ which are CCZ-equivalent but not EA-equivalent.

### Proof

As pointed out in Proposition 4, we can extend the examples for $p=2,3,5$ above to functions mapping into ${\mathbb {Z}}_{p^k}$, $k > 2$. Let *G*, *H*, *K*, *L* be any finite abelian groups. By [24, Theorem 1], there exist functions from $G\times K$ to $H\times L$, which are CCZ-equivalent but not EA-equivalent, if there exist functions from *G* to *H* which are CCZ-equivalent but not EA-equivalent. In particular, with $G = {\mathbb {F}}_{p^t}$, $K = {\mathbb {V}}_{n-t}^{(p)}$, where $t=2$ for $p=3,5$ and $t=4$ for $p=2$, and $H = {\mathbb {F}}_{p^k}$, $L = \{0\}$, we infer that there are functions from ${\mathbb {V}}_n^{(p)}$ to ${\mathbb {Z}}_{p^k}$ ($p=2,3,5$), which are CCZ-equivalent but not EA-equivalent. $\square$

### Remark 3

Note that more generally, with [24, Theorem 1] there exist functions from ${\mathbb {V}}_n^{(p)}\times K$ to ${\mathbb {Z}}_{p^k}\times L$, which are CCZ-equivalent but not EA-equivalent, for any finite abelian groups *K*, *L* when $p=2$, $n\ge 4$, $k\ge 2$, or $p=3,5$, $n\ge 2$, $k\ge 2$.

### Remark 4

We expect that it is difficult to show Theorem 3 for arbitrary primes *p*, using the same approach. It requires general results on the algebraic degree of $a_0$ and $a_1(\wp ^{-1})$ constructed as described above.

By Proposition 3 (iv), if $f(x) = a_0(x) + a_1(x)p + \cdots + a_{k-1}(x)p^{k-1}$ and *g* are CCZ-equivalent functions from ${\mathbb {V}}_n^{(p)}$ to ${\mathbb {Z}}_{p^k}$, and $a_0$ is bent, then *f* and *g* are EA-equivalent. We next extend this result to a much larger class of *p*-ary functions $a_0$.

### Theorem 4

Let $f:{\mathbb {V}}_n^{(p)}\rightarrow {\mathbb {Z}}_{p^k}$ be given as $f(x)=\sum _{i=0}^{k-1}a_i(x)p^i$ and suppose that the Walsh transform of $a_0$ satisfies $\mathcal {W}_{a_0}(b) \ne 0$ for all $b\in {\mathbb {V}}_n^{(p)}$. If *f* and *g* are CCZ-equivalent, then they are EA-equivalent.

### Proof

Let $f:{\mathbb {V}}_n^{(p)}\rightarrow {\mathbb {Z}}_{p^k}$ be given as $f(x) = \sum _{i=0}^{k-1}a_i(x)p^i$ and suppose that the Walsh transform of $a_0$ satisfies $\mathcal {W}_{a_0}(b) \ne 0$ for all $b\in {\mathbb {V}}_n^{(p)}$. Assume that *f* and *g* are CCZ-equivalent. Then there is an automorphism $\varphi (\mathcal {A},z,c,\gamma )$ with $\mathcal {A}$ being a linearized permutation of ${\mathbb {F}}_{p^n}$, $z, c\in {\mathbb {F}}_{p^n}, \gamma \in {\mathbb {Z}}_{p^k}^*$, such that we obtain the graph of *g*(*x*) after applying this automorphism to the graph of *f*(*x*). This is possible if and only if $\mathcal {A}(x)+za_0(x)$ is a permutation of ${\mathbb {F}}_{p^n}$. $\mathcal {A}(x)+za_0(x)$ is a permutation of ${\mathbb {F}}_{p^n}$ if and only if $\textrm{Tr}_n(\alpha (\mathcal {A}(x) + za_0(x)))$ is balanced for all nonzero $\alpha \in {\mathbb {F}}_{p^n}$ i.e., $\mathcal {W}_{\textrm{Tr}_n(\alpha (\mathcal {A}(x) + za_0(x)))}(0) = 0$ for any $\alpha \in {\mathbb {F}}_{p^n}^{*}$. The function $\textrm{Tr}_n(\alpha (\mathcal {A}(x) + za_0(x))) = \textrm{Tr}_n(\alpha (\mathcal {A}(x)))+\textrm{Tr}_n(\alpha z)a_0(x)$ is EA-equivalent to $a_0(x)$ unless $z=0$. Since the Walsh spectrum is preserved under EA-equivalence, we conclude that $\mathcal {W}_{\textrm{Tr}_n(\alpha (\mathcal {A}(x) + za_0(x)))}(0)$ is nonzero for all $\alpha \in {\mathbb {F}}_{p^n}^*$ unless $z=0$. $\square$

Several classes of *p*-ary functions studied in the literature, like non-bent plateaued functions, in particular non-bent quadratic functions, have 0’s in their Walsh spectrum. On the other hand, it is easily confirmed with random (non-quadratic) choices of *p*-ary functions, that many of them do not have 0 in the Walsh spectrum. Note that $a_0$ constructed as in Step 1 in the above procedure, must have 0 in its Walsh spectrum.

### Remark 5

Theorem 4 is of a similar flavour as Proposition 9 in [6], where an argument via the non-existence of zeros in the Walsh spectrum is used to confirm that for a vectorial bent function, CCZ-equivalence reduces to EA-equivalence, see also [4, Theorem 1]. The same argument does not apply to Proposition 3. It may be worthwhile to study the relation between Walsh zeros and equivalence in a general framework.

We close this section with a result on equivalence in connection with gbent functions.

### Proposition 5

Let $f:{\mathbb {V}}_n^{(p)}\rightarrow {\mathbb {Z}}_{p^k}$, $f(x) = a_0(x) + a_1(x)p + \cdots + a_{k-1}(x)p^{k-1}$ be a gbent function, and let $K:{\mathbb {V}}_n^{(p)}\rightarrow {\mathbb {F}}_p^{k-1}$ be given as $K(x) = (a_0(x),a_1(x),\ldots ,a_{k-2}(x))$. For some arbitrary functions $G:{\mathbb {F}}_p^{k-1}\rightarrow {\mathbb {Z}}_{p^{k-1}}$, $F:{\mathbb {F}}_p^{k-1}\rightarrow {\mathbb {F}}_p$, let

$$\begin{aligned} g(x)&= G(a_0(x),a_1(x),\ldots ,a_{k-2}(x)) + p^{k-1}(a_{k-1}(x) + F(a_0(x),a_1(x),\ldots ,a_{k-2}(x))) \\&= G(K(x)) + p^{k-1}(a_{k-1}(x) + F(K(x))). \end{aligned}$$

(i): *g* is a gbent function from ${\mathbb {V}}_n^{(p)}$ to ${\mathbb {Z}}_{p^k}$.
(ii): Let $g(x) = b_0(x) + b_1(x)p + \cdots + b_{k-1}(x)p^{k-1}$, then for the affine bent function spaces $\mathcal {A}(a_{k-1},\mathcal {P}^f_{a_{k-1}})$ and $\mathcal {A}(b_{k-1},\mathcal {P}^g_{b_{k-1}})$ we have $\mathcal {A}(a_{k-1},\mathcal {P}^f_{a_{k-1}})= \mathcal {A}(b_{k-1},\mathcal {P}^g_{b_{k-1}})$ if and only if *G* restricted to the image set *im*(*K*) of *K* is injective. In particular, if *G* is one-to-one, then $\mathcal {A}(a_{k-1},\mathcal {P}^f_{a_{k-1}}) = \mathcal {A}(b_{k-1},\mathcal {P}^g_{b_{k-1}})$.

### Proof

Clearly, *g* is a function from ${\mathbb {V}}_n^{p)}$ to ${\mathbb {Z}}_{p^k}$, hence we can write *g* as

$$\begin{aligned} g(x) = b_0(x) + b_1(x)p + \cdots + b_{k-1}(x)p^{k-1} \end{aligned}$$

for uniquely given *p*-ary functions $b_i$, $0\le i\le k-1$, where $b_{k-1}(x) = a_{k-1}(x) + F(a_0(x),a_1(x),\ldots ,a_{k-2}(x))$. Observe that for a function $\tilde{F}:{\mathbb {F}}_p^{k-1}\rightarrow {\mathbb {F}}_p$ we have

$$\begin{aligned}&b_{k-1}(x) + \tilde{F}(a_0(x),\ldots , a_{k-2}(x)) = a_{k-1}(x) + F(a_0(x),a_1(x),\ldots ,a_{k-2}(x)) + \\&\tilde{F}(a_0(x),\ldots , a_{k-2}(x)) = a_{k-1}(x) + \bar{F}(a_0(x),a_1(x),\ldots ,a_{k-2}(x)) \end{aligned}$$

for some $\bar{F}:{\mathbb {F}}_p^{k-1}\rightarrow {\mathbb {F}}_p$. Therefore $b_{k-1}(x) + \tilde{F}(a_0(x),\ldots , a_{k-2}(x))$ is a bent function, and we conclude that $b_{k-1}$ is admissible relative to $\mathcal {P}_{a_{k-1}}^f$. As easily seen, the sets in the partition $\mathcal {P}^g_{b_{k-1}}$ are unions of the sets of the partition $\mathcal {P}^f_{a_{k-1}}$. As a consequence, every *p*-ary bent function which is admissible relative to $\mathcal {P}^f_{a_{k-1}}$ is also admissible relative to $\mathcal {P}^g_{b_{k-1}}$. In particular, $b_{k-1}$ is admissible relative to $\mathcal {P}^g_{b_{k-1}}$, and *g* is gbent, which shows (i).

To show (ii), we first note that $\mathcal {P}_{a_{k-1}}^f$ respectively $\mathcal {P}_{b_{k-1}}^g$ is the preimage set partition of *K* respectively of *G*(*K*(*x*)), i.e. for every element in the image set *im*(*K*) of *K* respectively of *im*(*G*(*K*)), we have one set in $\mathcal {P}_{a_{k-1}}^f$ respectively in $\mathcal {P}_{b_{k-1}}^g$. Observing that $im(G(K)) \subseteq im(K)$ and $im(G(K)) = im(K)$ if and only if *G* is injective on *im*(*K*), shows the claim. $\square$

We say that $f(x) = a_0(x) + a_1(x)p + \cdots + a_{k-1}(x)p^{k-1}$ and $g(x) = b_0(x) + b_1(x)p + \cdots + b_{k-1}(x)p^{k-1}$ are *partition equivalent* if $\mathcal {A}(a_{k-1},\mathcal {P}^f_{a_{k-1}}) = \mathcal {A}(b_{k-1},\mathcal {P}^g_{b_{k-1}})$. Partition equivalence is an interesting concept for the case of gbent functions. If *f*, *g* are not gbent, this concept is less meaningful, as then in general $\mathcal {H}_f$ and $\mathcal {H}_g$ take on different values.

As one may expect, in general, *f*, *g* given as in Proposition 5 are not CCZ-equivalent. We confirm this fact with the following example.

### Example 4

$f(x) = a_0(x)+3a_1(x) = \textrm{Tr}^4_1(x) + 3\textrm{Tr}^4_1(x^{14})$ is a generalized bent function from ${\mathbb {F}}_{3^4}$ to ${\mathbb {Z}}_{3^2}$. $a_1(x)$ is a Coulter-Matthews bent function. Consequently, *f* has the six bent components $a_1, 2a_1, a_0+a_1, 2a_0+a_1, a_0+2a_1, 2a_0+2a_1$. Let $g(x) = G(a_0(x)) + 3(a_1(x) + F(a_0(x)) = b_0(x) + 3b_1(x)$, where $F,G:{\mathbb {F}}_3\rightarrow {\mathbb {F}}_3$ are given as $G(x) = x^2$ and $F(x) = 0$. Then the Walsh spectra of the components of *g* are given as follows:

**Table:** 

Component	Walsh transform
$b_0$	$0^{78}, (-27\zeta _3 + 27)^2, 54\zeta _3 + 27$
$2b_0$	$0^{78}, -54\zeta _3 - 27, (27\zeta _3 + 54)^{2}$
$b_1$	$-9^{21},-9\zeta _3^{30}, (9\zeta _3 + 9)^{30}$
$2b_1$	$-9^{21},-9\zeta _3^{30}, (9\zeta _3 + 9)^{30}$
$b_0+b_1$	$0^{24}, -9^{8}, 9, (-9\zeta _3 - 9)^{4}, -9\zeta _3^{20}, (9\zeta _3 - 9)^{4}, 9\zeta _3^4, (9\zeta _3 + 9)^8, (9\zeta _3 + 18)^8$
$2b_0+b_1$	$0^{6}, -9^{8}, 9^{16}, (-9\zeta _3 - 18)^{2}, (-9\zeta _3 - 9)^4, -9\zeta _3^{20}, 9\zeta _3^{16}, (9\zeta _3 + 9)^8, 18\zeta _3 + 9$
$b_0+2b_1$	$0^{6}, -9^{8}, 9^{16}, -18\zeta _3 - 9, (-9\zeta _3 - 9)^{16}, -9\zeta _3^8, (9\zeta _3 - 9)^2, 9\zeta _3^4, (9\zeta _3 + 9)^{20}$
$2b_0+2b_1$	$0^{24}, -9^{8}, 9, (-9\zeta _3 - 18)^4, (-9\zeta _3 - 9)^4, -9\zeta _3^8, (-9\zeta _3 + 9)^8, 9\zeta _3^4, (9\zeta _3 + 9)^{20}$

Since *f* has six bent components, whereas *g* has only two, $b_1$ and $2b_1$, *f* and *g* are not EA-equivalent. Because $a_0(x) = \textrm{Tr}^4_1(x)$ is linear, by Proposition 3, *f* and *g* are also not CCZ-equivalent.

## Constructions of generalized bent functions

By the fundamental result in Theorem 2, we can see a generalized bent function from ${\mathbb {V}}_n^{(p)}$ to ${\mathbb {Z}}_{p^k}$, *p* odd or $p=2$ and *n* even, as a bent function $a:{\mathbb {V}}_n^{(p)}\rightarrow {\mathbb {F}}_p$ which is admissible relative to a partition $\mathcal {P}_a$ of ${\mathbb {V}}_n^{(p)}$. As described in Section 2, this gives rise to an affine space of bent functions $\mathcal {A}(a,\mathcal {P}_a)$ of dimension $|\mathcal {P}_a|$. Maiorana-McFarland bent functions are admissible relative to largest possible partitions, for which $|\mathcal {P}_a| = p^{n/2}$. Other functions which are admissible relative to a large partition are bent functions constructed from spreads. We refer to [22]. In [19], further generalized bent functions with large dimension are constructed using the secondary generalized Maiorana-McFarland construction.

In this section some other secondary constructions, namely direct sum and semi-direct sum, and a secondary construction which we infer from [1, 28], are employed to construct generalized bent functions with large dimension. Finally, employing the generalized Maiorana-McFarland construction, we present a solution for constructing generalized bent functions from ${\mathbb {V}}_n^{(2)}$ to ${\mathbb {Z}}_{2^k}$ when *n* is odd.

### Direct sum and semi-direct sum

Let $a:{\mathbb {V}}_m^{(p)}\rightarrow {\mathbb {F}}_p$ and $b:{\mathbb {V}}_n^{(p)}\rightarrow {\mathbb {F}}_p$ be bent functions, then the *direct sum* $a(x) + b(y)$ is a bent function from ${\mathbb {V}}_m^{(p)}\times {\mathbb {V}}_n^{(p)}$ to ${\mathbb {F}}_p$. For the Walsh transform we have $\mathcal {W}_{a+b}(u,v) = \mathcal {W}_a(u)\mathcal {W}_b(v)$. Similarly, for generalized *p*-ary functions *f* from ${\mathbb {V}}_m^{(p)}$ to ${\mathbb {Z}}_{p^k}$, *g* from ${\mathbb {V}}_n^{(p)}$ to ${\mathbb {Z}}_{p^k}$ and $f(x)+g(y)$ from ${\mathbb {V}}_m^{(p)}\times {\mathbb {V}}_n^{(p)}$ to ${\mathbb {Z}}_{p^k}$, we have $\mathcal {H}_{f+g}(u,v) = \mathcal {H}_f(u)\mathcal {H}_g(v)$, and $f(x)+g(y)$ is gbent if *f* and *g* are gbent.

In [12], a generalization of the direct sum, called the *semi-direct sum* of bent functions is introduced: Let $a:{\mathbb {V}}_m^{(p)}\rightarrow {\mathbb {F}}_p$ and $b:{\mathbb {V}}_n^{(p)}\rightarrow {\mathbb {F}}_p$ be bent, and let $\varphi$ be a function from ${\mathbb {V}}_m^{(p)}$ to ${\mathbb {V}}_n^{(p)}$ such that $\Psi _\beta : {\mathbb {V}}_m^{(p)} \rightarrow {\mathbb {F}}_p$, $\Psi _\beta (x) = a(x) + \langle \beta , \varphi (x) \rangle _n$ is a bent function for all $\beta \in {\mathbb {V}}_n^{(p)}$. Then

8 $$\begin{aligned} h(x, y) = a(x) + b(y + \varphi (x)) \end{aligned}$$

is a bent function from ${\mathbb {V}}_m^{(p)}\times {\mathbb {V}}_n^{(p)}$ to ${\mathbb {F}}_p$. Note that if $\varphi = 0$, then all conditions are satisfied, and the semi-direct sum in (8) reduces to the direct sum.

#### Direct sum

Let $f(x) = a_0(x) + pa_1(x) + \cdots + p^{k-1}a_{k-1}(x)$ and $g(x) = b_0(x) + pb_1(x) + \cdots + p^{k-1}b_{k-1}(x)$ be gbent functions from ${\mathbb {V}}_m^{(p)}$ to ${\mathbb {Z}}_{p^k}$ and from ${\mathbb {V}}_n^{(p)}$ to ${\mathbb {Z}}_{p^k}$. By Theorem 2, we identify *f*, *g* with $(a_{k-1},\mathcal {P}^f_{a_{k-1}})$ and $(b_{k-1},\mathcal {P}^g_{b_{k-1}})$. We may choose two different approaches.

(i): Form the direct sum $c(x,y) = a_{k-1}(x) + b_{k-1}(y)$ and determine a partition $\mathcal {P}_c$ of ${\mathbb {V}}_m^{(p)}\times {\mathbb {V}}_n^{(p)}$ relative to which *c*(*x*, *y*) is admissible, in terms of the partitions $\mathcal {P}^f_{a_{k-1}}$ of ${\mathbb {V}}_m^{(p)}$ and $\mathcal {P}^g_{b_{k-1}}$ of ${\mathbb {V}}_n^{(p)}$.
(ii): Apply the direct sum to *f*, *g*, i.e., form the gbent function $f(x) + g(y)$, which we identify with $(c,\mathcal {P}_c)$ for an appropriate bent function $c:{\mathbb {V}}_m^{(p)}\times {\mathbb {V}}_n^{(p)}\rightarrow {\mathbb {F}}_p$ and a partition $\mathcal {P}_c$ of ${\mathbb {V}}_m^{(p)}\times {\mathbb {V}}_n^{(p)}$ relative to which *c* is admissible. Determine the bent function *c* in terms of *f*, *g* and $\mathcal {P}_c$ in terms of $\mathcal {P}^f_{a_{k-1}}$ and $\mathcal {P}^g_{b_{k-1}}$.

The first approach seems not very promising. It seems that in general, the direct sum of two bent functions *a*, *b* with (large) partitions $\mathcal {P}^f_a$, $\mathcal {P}^g_b$ is not admissible relative to an interesting partition.

We apply approach (ii). Recall that for the gbent functions $f:{\mathbb {V}}_m^{(p)}\rightarrow {\mathbb {Z}}_{p^k}$ and $g:{\mathbb {V}}_n^{(p)}\rightarrow {\mathbb {Z}}_{p^k}$ given as above, the partitions $\mathcal {P}^f_{a_{k-1}}$ and $\mathcal {P}^g_{b_{k-1}}$ are given by

$$\begin{aligned} \mathcal {P}^f_{a_{k-1}}&= \{P_0,P_1\ldots ,P_{p^{k-1}-1}\},\,\text{ where }\, P_j = \{x\in {\mathbb {V}}_m^{(p)}\,:\,f(x) = j \bmod p^{k-1}\} \\ \mathcal {P}^g_{b_{k-1}}&= \{Q_0,Q_1\ldots ,Q_{p^{k-1}-1}\},\,\text{ where }\, Q_j = \{x\in {\mathbb {V}}_n^{(p)}\,:\,g(x) = j \bmod p^{k-1}\}. \end{aligned}$$

Note that some of the sets $P_j,Q_j$ may be empty.

#### Theorem 5

Let $f:{\mathbb {V}}_m^{(p)}\rightarrow {\mathbb {Z}}_{p^k}$ and $g:{\mathbb {V}}_n^{(p)}\rightarrow {\mathbb {Z}}_{p^k}$ be gbent functions with partitions $\mathcal {P}^f_{a_{k-1}}$ and $\mathcal {P}^g_{b_{k-1}}$ given as above. The function $c:{\mathbb {V}}_m^{(p)}\times {\mathbb {V}}_n^{(p)}\rightarrow {\mathbb {F}}_p$ given by

$$\begin{aligned} c(x,y) = r\,\text{ if }\; (f(x) + g(y)) \bmod p^k \in \{rp^{k-1}, rp^{k-1}+1,\ldots , (r+1)p^{k-1}-1\} \end{aligned}$$

is a bent function, which is admissible relative to the partition $\mathcal {P}_c=\{R_1,\ldots ,R_{p^{k-1}-1}\}$ of ${\mathbb {V}}_m^{(p)}\times {\mathbb {V}}_n^{(p)}$, where for $0\le j\le p^{k-1}-1$,

9 $$\begin{aligned} R_j = \bigcup _{i=0}^{p^{k-1}-1}P_{i}\times Q_{j-i} \quad \text{(indices } \text{ are } \text{ determined } \text{ modulo } p^{k-1}\text{) }. \end{aligned}$$

A representation of $(c,\mathcal {P}_c)$ is the direct sum $h(x,y) = f(x) + g(y) = \sum _{i=0}^{k-1}c_i(x,y)p^i$, for which $c_{k-1}(x,y) = c(x,y)$.

(Note that $P_i\times Q_{j-i}$ is the empty set if $P_i$ or $Q_{j-i}$ is the empty set.)

#### Proof

The direct sum $h:{\mathbb {V}}_m^{(p)}\times {\mathbb {V}}_n^{(p)}\rightarrow {\mathbb {Z}}_{p^k}$ of *f* and *g*,

$$\begin{aligned} h(x,y) = f(x) + g(y) = c_0(x,y) + c_1(x,y)p + \cdots + c_{k-1}(x,y)p^{k-1} \end{aligned}$$

is a gbent function. Consequently, $c_{k-1} = c$ is a bent function from ${\mathbb {V}}_m^{(p)}\times {\mathbb {V}}_n^{(p)}$ to ${\mathbb {F}}_p$, which is admissible relative to the partition of $\mathcal {P}^h_c = \{R_1,\ldots ,R_{p^{k-1}-1}\}$ of ${\mathbb {V}}_m^{(p)}\times {\mathbb {V}}_n^{(p)}$, where

$$\begin{aligned} R_j = \{(x,y)\in {\mathbb {V}}_m^{(p)}\times {\mathbb {V}}_n^{(p)}\;:\; h(x,y) = f(x) + g(y) = j \bmod p^{k-1}\}. \end{aligned}$$

As easily seen, $f(x) + g(y) = j \bmod p^{k-1}$ if and only if $x\in P_i$ and $y\in Q_{j-i}$ for some $0\le i\le p^{k-1}-1$ (indices determined modulo $p^{k-1}$). Observing that $c_{k-1}(x,y)$ equals the coefficient of $p^{k-1}$ in the *p*-ary representation of $f(x) + g(y)$, yields the description of $c_{k-1}(x,y) = c(x,y)$ as given in the theorem. $\square$

#### Remark 6

In general, $c(x,y) \ne a_{k-1}(x)+b_{k-1}(y)$, and $a_{k-1}(x)+b_{k-1}(y)$ is not admissible relative to the partition in (9). If $g(x) = p^{k-1}b(x)$, which has the trivial partition $\mathcal {P}^g_b = \{Q_0 = {\mathbb {V}}_n^{(p)}\}$, then $c(x,y) = a_{k-1}(x)+b_{k-1}(y)$, and $R_j = P_j\times {\mathbb {V}}_n^{(p)}$.

#### Remark 7

Choosing other representations $\tilde{f},\tilde{g}$ of $(a_{k-1},\mathcal {P}^f_{a_{k-1}})$, $(b_{k-1},\mathcal {P}^g_{b_{k-1}})$ described as in Proposition 5, gives a description of $\mathcal {P}^f_{a_{k-1}} = \{P_0, P_1, \ldots , P_{p^{k-1}-1}\}$ and $\mathcal {P}^g_{b_{k-1}} = \{Q_0, Q_1, \ldots , Q_{p^{k-1}-1}\}$ as $\mathcal {P}^f_{a_{k-1}} = \{P_{\tau _1(0)}, P_{\tau _1(1)}, \ldots , P_{\tau _1(p^{k-1}-1)}\}$ and $\mathcal {P}^g_{b_{k-1}} = \{Q_{\tau _2(0)}, Q_{\tau _2(1)}, \ldots , Q_{\tau _2(p^{k-1}-1)}\}$ for some permutations $\tau _1, \tau _2$ of $\{0, 1, \ldots , p^{k-1}-1\}$. In general, the bent function *c* and the partition $\mathcal {P}_c$ constructed in Theorem 5 will then be different. With the direct sum, we therefore can obtain various gbent functions $(c,\mathcal {P}_c)$ from $(a_{k-1},\mathcal {P}^f_{a_{k-1}})$ and $(b_{k-1},\mathcal {P}^g_{b_{k-1}})$.

#### Semi-direct sum

As for the direct sum, we consider a version of the semi-direct sum for generalized *p*-ary functions. We will use the following lemma.

#### Lemma 1

Let $f(x) = a_0(x) + a_1(x)p + \cdots + a_{k-1}(x)p^{k-1}$ be a gbent function and let $\varphi :{\mathbb {V}}_m^{(p)}\rightarrow {\mathbb {V}}_n^{(p)}$ be constant on the sets of the partition $\mathcal {P}^f_{a_{k-1}}$ of *f*. Then for every $\beta \in {\mathbb {V}}_n^{(p)}$, the function $a_{k-1}(x) + \langle \beta ,\varphi (x)\rangle _n$ is a bent function, which is admissible relative to $\mathcal {P}^f_{a_{k-1}}$. Hence

$$\begin{aligned} f_\beta (x) = a_0(x) + a_1(x)p + \cdots + (a_{k-1}(x)+\langle \beta ,\varphi (x)\rangle _n)p^{k-1} \end{aligned}$$

is a gbent function.

#### Proof

Since $\varphi$ is constant on the sets of the partition $\mathcal {P}^f_{a_{k-1}}$ we have

$$\begin{aligned} a_{k-1}(x) + \langle \beta ,\varphi (x)\rangle _n = a_{k-1}(x) + C_{\varphi ,\beta }(x) \end{aligned}$$

for a *p*-ary function $C_{\varphi ,\beta }$ which is constant on the sets of $\mathcal {P}^f_{a_{k-1}}$. By Theorem 2, $a_{k-1}(x) + \langle \beta ,\varphi (x)\rangle _n$ is a bent function, and it is admissible relative to the same partition $\mathcal {P}^f_{a_{k-1}}$. $\square$

#### Proposition 6

For $f:{\mathbb {V}}_m^{(p)}\rightarrow {\mathbb {Z}}_{p^k}$, $g:{\mathbb {V}}_n^{(p)}\rightarrow {\mathbb {Z}}_{p^k}$ and $\varphi :{\mathbb {V}}_m^{(p)}\rightarrow {\mathbb {V}}_n^{(p)}$ consider the semi-direct sum

$$\begin{aligned} h(x,y) = f(x) + g(y - \varphi (x)). \end{aligned}$$

If *f* and *g* are gbent functions, and $\varphi$ is constant on the sets of the partition $\mathcal {P}^f_{a_{k-1}}$ of the gbent function *f*, then *h* is a gbent function from ${\mathbb {V}}_m^{(p)}\times {\mathbb {V}}_n^{(p)}$ to ${\mathbb {Z}}_{p^k}$.

#### Proof

We show that for $\alpha \in {\mathbb {V}}_m^{(p)}$, $\beta \in {\mathbb {V}}_n^{(p)}$ we have $\mathcal {H}_h(\alpha ,\beta ) = \mathcal {H}_{f_\beta }(\alpha )\mathcal {H}_g(\beta )$. With Lemma 1, $f_\beta$ is gbent for all $\beta \in {\mathbb {V}}_n^{(p)}$, which completes the proof. For simplicity writing the inner product as $u\cdot v$, we have

$$\begin{aligned} \mathcal {H}_h(\alpha ,\beta )&= \underset{\underset{y\in {\mathbb {V}}_n^{(p)}}{x\in {\mathbb {V}}_m^{(p)}}}{\sum } \zeta _{p^k}^{f(x)+g(y-\varphi (x))} \zeta _p^{\alpha \cdot x + \beta \cdot y} = \sum _{x\in {\mathbb {V}}_m^{(p)}}\zeta _{p^k}^{f(x)}\zeta _p^{\alpha \cdot x} \sum _{y\in {\mathbb {V}}_n^{(p)}}\zeta _{p^k}^{g(y-\varphi (x))}\zeta _p^{\beta \cdot y} \\&= \sum _{x\in {\mathbb {V}}_m^{(p)}}\zeta _{p^k}^{f(x)}\zeta _p^{\alpha \cdot x} \sum _{y\in {\mathbb {V}}_n^{(p)}}\zeta _{p^k}^{g(y)}\zeta _p^{\beta \cdot (y+\varphi (x))} = \sum _{x\in {\mathbb {V}}_m^{(p)}}\zeta _{p^k}^{f(x)}\zeta _p^{\alpha \cdot x + \beta \cdot \varphi (x)} \sum _{y\in {\mathbb {V}}_n^{(p)}}\zeta _{p^k}^{g(y)}\zeta _p^{\beta \cdot y} \\&= \sum _{x\in {\mathbb {V}}_m^{(p)}}\zeta _{p^k}^{f_\beta (x)}\zeta _p^{\alpha \cdot x} \sum _{y\in {\mathbb {V}}_n^{(p)}}\zeta _{p^k}^{g(y)}\zeta _p^{\beta \cdot y} = \mathcal {H}_{f_\beta }(\alpha )\mathcal {H}_g(\beta ), \end{aligned}$$

where in the last step we use that $\zeta _p^{\beta \cdot \varphi (x)} = \zeta _{p^k}^{p^{k-1}(\beta \cdot \varphi (x))}$. $\square$

#### Theorem 6

Let $f:{\mathbb {V}}_m^{(p)}\rightarrow {\mathbb {Z}}_{p^k}$ and $g:{\mathbb {V}}_n^{(p)}\rightarrow {\mathbb {Z}}_{p^k}$ be gbent functions with partitions $\mathcal {P}^f_{a_{k-1}}$ and $\mathcal {P}^g_{b_{k-1}}$ given as above. Let $\varphi :{\mathbb {V}}_m^{(p)}\rightarrow {\mathbb {V}}_n^{(p)}$ be any function constant on the sets of the partition $\mathcal {P}^f_{a_{k-1}}$, say $\varphi (P_i) = \gamma _i$. The function $c:{\mathbb {V}}_m^{(p)}\times {\mathbb {V}}_n^{(p)}\rightarrow {\mathbb {F}}_p$ given by

$$\begin{aligned} c(x,y) = r \;\text{ if }\; (f(x) + (g(y)-\varphi (x))) \bmod p^k \in \{rp^{k-1}, rp^{k-1}+1,\ldots , (r+1)p^{k-1}-1\} \end{aligned}$$

is a bent function, and admissible relative to the partition $\{R_1,\ldots ,R_{p^{k-1}-1}\}$ of ${\mathbb {V}}_m^{(p)}\times {\mathbb {V}}_n^{(p)}$, where for $0\le j\le p^{k-1}-1$,

$$\begin{aligned} R_j = \bigcup _{i\in {\mathbb {Z}}_{p^{k-1}}}(P_i \times (Q_{j-i} + c_i))\quad \text{(indices } \text{ determined } \text{ modulo } p^{k-1}\text{) }. \end{aligned}$$

A representation of $(c,\mathcal {P}_c)$ is the semi-direct sum $h(x,y) = f(x) + g(y-\varphi (x)) = \sum _{i=0}^{k-1}c_i(x,y)p^i$, for which $c_{k-1} = c$.

#### Proof

With our assumptions, by Proposition 6, the semi-direct sum

$$\begin{aligned} h(x,y) = f(x) + g(y - \varphi (x)) = c_0(x,y) + c_1(x,y)p + \cdots + c_{k-1}(x,y)p^{k-1} \end{aligned}$$

represents a gbent function from ${\mathbb {V}}_m^{(p)}\times {\mathbb {V}}_n^{[(p)}$ to ${\mathbb {Z}}_{p^k}$. The description of the bent function $c_{k-1} = c$ and the expression of the partition $\mathcal {P}^h_c$ in terms of the partitions $\mathcal {P}^f_{a_{k-1}}$ and $\mathcal {P}^g_{b_{k-1}}$ we then obtain in the same way as for the direct sum in Theorem 5. $\square$

### The construction in [1, 28]

In [28, Theorem 4], a secondary construction of vectorial bent functions is introduced, which then has been refined in Section 5 in [1]. This construction can be employed to generate, as a special case, *p*-ary bent functions *h* from ${\mathbb {V}}_n^{(p)}\times {\mathbb {V}}_{2m}^{(p)}$ to ${\mathbb {F}}_p$ from *p* bent functions from ${\mathbb {V}}_n^{(p)}$ to ${\mathbb {F}}_p$, see Proposition 7 below. For this construction we need the concept of vectorial dual-bent functions.

Let $F:{\mathbb {V}}_n^{(p)}\rightarrow {\mathbb {V}}_k^{(p)}$ be a vectorial bent function, then the *component functions* $F_c(x) = \langle c,F(x)\rangle _k$, $c\in {\mathbb {V}}_k^{(p)}$ nonzero, form a *k*-dimensional vector space of *p*-ary bent functions (together with the 0-function). A vectorial bent function $F:{\mathbb {V}}_n^{(p)}\rightarrow {\mathbb {V}}_k^{(p)}$ is called a *vectorial dual-bent function* if the set of the duals of its component functions,

$$\begin{aligned} \{(F_c)^* \,:\,c\in {\mathbb {V}}_k^{(p)},\,\text{ nonzero }\}, \end{aligned}$$

also forms a *k*-dimensional vector space of bent functions. The set of the duals are then the components of another vectorial bent function $F^*:{\mathbb {V}}_n^{(p)}\rightarrow {\mathbb {V}}_k^{(p)}$, called *a vectorial dual of F*. We refer to [11], where this concept has been introduced.

Let $F:{\mathbb {V}}_n^{(p)}\rightarrow {\mathbb {V}}_k^{(p)}$ be a vectorial dual-bent function with a vectorial dual $F^*$, then for the components of *F*, $F^*$ we have

10 $$\begin{aligned} F_c^* = (F_{\sigma (c)})^* \quad \text{ for } \text{ some } \text{ permutation } \sigma \text{ of } {\mathbb {V}}_k^{(p)}\setminus \{0\}. \end{aligned}$$

We follow the notation in [28] and say that a vectorial dual-bent function $F:{\mathbb {V}}_n^{(p)}\rightarrow {\mathbb {V}}_k^{(p)}$ satisfies Condition A, if

- *F* has a vectorial dual $F^*$ for which $\sigma$ in (10) is the identity permutation, and
- all components of *F* are regular or all are weakly regular but not regular.

We can now state a *p*-ary version of the construction in [1, 28]. We include a proof for the convenience of the reader.

#### Proposition 7

Let *n*, $m\le n/2$ and $1<k\le m$ be integers, and let $\alpha ,\beta$ be elements of ${\mathbb {F}}_{p^k}$ which are linearly independent over ${\mathbb {F}}_p$. Let $e(y):{\mathbb {V}}_{2m}^{(p)}\mapsto {\mathbb {F}}_{p^k}$ be a vectorial dual-bent function satisfying Condition A, and for $i\in {\mathbb {F}}_p$, let *f*(*i*; *x*) be a bent function from ${\mathbb {V}}_n^{(p)}$ to ${\mathbb {F}}_p$. Then $h:{\mathbb {V}}_n^{(p)}\times {\mathbb {V}}_{2m}^{(p)}\rightarrow {\mathbb {F}}_p$,

11 $$\begin{aligned} h(x,y) = f(\textrm{Tr}_{1}^{k} (\alpha e(y));x)+\textrm{Tr}_1^{k} (\beta e(y)) \end{aligned}$$

is a *p*-ary bent function.

#### Proof

Consider the component functions $l,t:{\mathbb {V}}_{2m}^{(p)}\rightarrow {\mathbb {F}}_p$ of *e*(*y*),

$$\begin{aligned} \ell (y) = e_\alpha (y) = \textrm{Tr}_1^k(\alpha e(y)) \; \, \text {and } \;\, t(y) = e_\beta (y) = \textrm{Tr}_1^k(\beta e(y)), \end{aligned}$$

and note that since $\alpha ,\beta$ are linearly independent over ${\mathbb {F}}_p$, for every $j\in {\mathbb {F}}_p$, $t(y) - j\ell (y) = e_{\beta -j\alpha }(y)$ is a bent component of *e*(*y*) as well. Since *e*(*y*) satisfies Condition A, i.e., *e*(*x*) has a vectorial dual $e^*$ such that for every component we have $(e_c)^* = e^*_c$, for every $j\in {\mathbb {F}}_p$ and $b\in {\mathbb {V}}_{2m}^{(p)}$ we have

12 $$\begin{aligned} \mathcal {W}_{t-j\ell }(b) = \mathcal {W}_{e_{\beta -j \alpha }}(b) = p^m \zeta _p^{e^*_{\beta -j \alpha }(b)}. \end{aligned}$$

(We here suppose that all components of *e* are regular, it is analogous if all components are weakly regular but not regular.) Then for $a\in {\mathbb {V}}_n^{(p)}$ and $b\in {\mathbb {V}}_{2m}^{(p)}$, we have

$$\begin{aligned} \mathcal {W}_h(a,b)&= \sum _{i\in {\mathbb {F}}_p}\sum _{x\in \mathbb {V}_n^{(p)}} \sum _{y\in {\mathbb {V}}_{2m}^{(p)} : \ell (y)=i} \zeta _p^{f(i;x) + t(y) -<a,x>_n-<b,y>_{2m}} \\&=\sum _{i\in {\mathbb {F}}_p} \sum _{x\in \mathbb {V}_n^{(p)}}\zeta _p^{f(i;x)-<a,x>_n } \sum _{y\in {\mathbb {V}}_{2m}^{(p)}: \ell (y)=i} \zeta _p^{t(y)-<b,y>_{2m}}\\&=p^{-1}\sum _{i\in {\mathbb {F}}_p}\mathcal {W}_{f(i;x)}(a) \sum _{y\in {\mathbb {V}}_{2m}^{(p)}}\zeta _p^{t(y)-<b,y>_{2m}} \sum _{j\in {\mathbb {F}}_p} \zeta _p^{j(i-\ell (y)) }\\&= p^{-1}\sum _{i\in {\mathbb {F}}_p} \mathcal {W}_{f(i;x)}(a) \sum _{j\in {\mathbb {F}}_p} \zeta _p^{ij } \mathcal {W}_{t-j\ell }(b) . \end{aligned}$$

Then by (12), we have the following equalities.

$$\begin{aligned} \mathcal {W}_h(a,b)&= p^{m-1} \sum _{i\in {\mathbb {F}}_p}\mathcal {W}_{f(i;x)}(a) \sum _{j\in {\mathbb {F}}_p} \zeta _p^{ij}\zeta _p^{e^*_{\beta -j \alpha }(b)}\\&= p^{m-1} \sum _{i\in {\mathbb {F}}_p}\mathcal {W}_{f(i;x)}(a) \sum _{j\in {\mathbb {F}}_p} \zeta _p^{ij}\zeta _p^{\textrm{Tr}_1^k((\beta -j \alpha ) e^*(b))} \\&= p^{m-1} \zeta _p^{ \textrm{Tr}_{1}^{k}(\beta e^*(b)) } \sum _{i\in {\mathbb {F}}_p}\mathcal {W}_{f(i;x)}(a) \sum _{j\in {\mathbb {F}}_p}\zeta _p^{j(i-\textrm{Tr}_{1}^{k} (\alpha e^*(b) )) }\\&= p^{m} \zeta _p^{\textrm{Tr}_{1}^{k}(\beta e^*(b) ) } \mathcal {W}_{f(\textrm{Tr}_{1}^{k} (\alpha e^*(b) );x)}(a) = p^{n+m} \zeta _p^{J(a,b)}, \end{aligned}$$

where $$J(a,b) = \textrm{Tr}_{1}^{k}(\beta e^*(b) ) + (f(\textrm{Tr}_{1}^{k} (\alpha e^*(b) );x))^*(a)$$.$$\square $$

#### Remark 8

Examples of vectorial dual-bent functions *e*(*y*) satisfying Condition A can be obtained from generalized semifield spreads [2]. Secondary constructions are in [1] and in [28], where a vectorial version of the construction in Proposition 7 is employed for this purpose. We also remark that all so far constructed vectorial dual-bent functions satisfying Condition A have regular components.

Similar as for the direct and for the semi-direct sum, we employ the construction of a bent function in Proposition 7 for constructing a gbent function. Equivalently, we construct a (large) affine space $\mathcal {A}(h,\mathcal {P}_h)$ of bent functions from ${\mathbb {V}}_{n+2m}^{(p)}$ to ${\mathbb {F}}_p$, from (large) affine spaces $\mathcal {A}(f(i;x),\mathcal {P}_{f(i;x)})$ of bent functions from ${\mathbb {V}}_n^{(p)}$ to ${\mathbb {F}}_p$.

#### Theorem 7

Let *n*, $m\le n/2$ and $1<k\le m$ be integers, and let $\alpha ,\beta$ be elements of ${\mathbb {F}}_{p^k}$ which are linearly independent over ${\mathbb {F}}_p$. For a vectorial dual-bent function $e:{\mathbb {V}}_{2m}\rightarrow {\mathbb {F}}_{p^k}$ satisfying Condition A, and bent functions *f*(*i*; *x*), $i=0,\ldots , p-1$, from ${\mathbb {V}}_n^{(p)}$ to ${\mathbb {F}}_p$, let $h:{\mathbb {V}}_n^{(p)}\times {\mathbb {V}}_{2m}^{(p)}$ be the bent function

$$\begin{aligned} h(x,y) = f(\textrm{Tr}_{1}^{k} (\alpha e(y));x)+\textrm{Tr}_1^{k} (\beta e(y)). \end{aligned}$$

If for $i = 0,1,\ldots ,p-1$, *f*(*i*; *x*) is admissible relative to the partition $\mathcal {P}_i=\{P_1^{(i)}, \ldots , P_{r_i}^{(i)}\}$ of ${\mathbb {V}}_n^{(p)}$, then *h* is admissible relative to the partition

$$\begin{aligned} \mathcal {P}_h = \{P_j^{(i)} \times Q_i\,:\,0\le i\le p-1, 1\le j\le r_i\} \end{aligned}$$

of $${\mathbb {V}}_n^{(p)}\times {\mathbb {V}}_{2m}^{(p)}$$, where $$Q_i = \{y\in {\mathbb {V}}_{2m}^{(p)}\,:\,\textrm{Tr}_{1}^{k} (\alpha e(y)) = i\}$$.

#### Proof

First note that $\mathcal {P}_h$ defined as in the theorem, is indeed a partition of ${\mathbb {V}}_n^{(p)}\times {\mathbb {V}}_{2m}^{(p)}$. We have to show that for each $c_{ij} \in {\mathbb {F}}_p$, the function

$$\begin{aligned} \bar{h}(x,y) = h(x,y) + \sum _{i \in {\mathbb {F}}_p}\sum _{j=1}^{r_i}c_{ij}\phi _{P_j^{(i)}\times Q_i}(x,y) \end{aligned}$$

is a bent function.

By assumption, for each $i=0,1,\ldots , p-1$, the function $\widetilde{f}(i;x) = f(i;x) + \sum _{j=1}^{r_i} c_{ij}\phi _{P_j^{(i)}}(x)$ is a bent function for any choice of $c_{ij}\in {\mathbb {F}}_p$. Again we denote by $\ell (y)$ and *t*(*y*) the bent functions $\ell (y) = \textrm{Tr}_1^k(\alpha e(y))$ and $t(y) = \textrm{Tr}_1^k(\beta e(y))$. Applying the construction in Proposition 7 to the functions $\widetilde{f}(i;x)$, we then obtain the bent function

$$\begin{aligned} \widetilde{h}(x,y)&= \widetilde{f}(\ell (y);x) + t(y) \\&= f(\ell (y);x) + \sum _{j=1}^{r_{\ell (y)}} c_{\ell (y)j}\phi _{P_{j}^{(\ell (y))}}(x) + t(y) \\&= h(x,y) + \sum _{j=1}^{r_{\ell (y)}} c_{\ell (y)j}\phi _{P_{j}^{(\ell (y))}}(x). \end{aligned}$$

We finish the proof showing that $\widetilde{h}(x,y) = \bar{h}(x,y)$: Let $(x^{'},y^{'})$ be an arbitrary element of ${\mathbb {V}}_n^{(p)} \times {\mathbb {V}}_{2m}^{(p)}$. Let $i=\ell (y^{'})$ and $P_{\kappa }^{(\ell (y^{'}))}$ be the unique set that contains $x^{'}$. Then $\widetilde{h}(x^{'},y^{'}) = h(x^{'},y^{'}) + c_{\ell (y^{'})\kappa }$ and $\bar{h}(x^{'},y^{'}) = h(x^{'},y^{'}) + c_{\ell (y^{'})\kappa }$. $\square$

#### Remark 9

The affine bent function space $\mathcal {A}(h,\mathcal {P}_h)$ has dimension $r = \sum _{i=0}^{p-1}r_i$ if $\mathcal {A}(f(i;x),\mathcal {P}_{f(i;x)})$ has dimension $r_i$, $i=0,1,\ldots ,p-1$.

### Construction of gbent functions for $p=2$ and *n* is odd

The construction of gbent functions in [19], the direct and the semi-direct sum in Subsection 4.1, and the construction given in Subsection 4.2, can be applied whenever *p* is odd, or $p = 2$ and *n* is even. For the case that $p=2$ and *n* is odd, bent functions do not exist, and Theorem 2 does not apply.

In this section, we construct gbent functions for $p=2$ and *n* odd. We will have to consider functions in $n-1$ variables, in *n* variables and in $n+1$ variables. We therefore introduce the notation $(x) = (x_1,x_2,\ldots ,x_{n-1})$, $(X) = (x,y) = (x_1,x_2,\ldots ,x_{n-1},y)$ and $(x,y,z) = (X,z) = (x_1,x_2,\ldots ,x_{n-1},y,z)$.

We use the following connection between Boolean gbent functions in even and in odd dimension, given in [23].

#### Proposition 8

 [23, Theorem 24] For an odd integer *n*, let $f:{\mathbb {V}}_n^{(2)}\rightarrow {\mathbb {Z}}_{2^k}$ be given as

$$\begin{aligned} f(X) = a_0(X) + 2a_1(X) + \cdots + 2^{k-2}a_{k-2}(X) + 2^{k-1}a_{k-1}(X) \end{aligned}$$

for some Boolean functions $a_i:{\mathbb {V}}_n^{(2)}\rightarrow {\mathbb {F}}_2$, $i=0,\ldots ,k-1$.

Then *f* is gbent if and only if $\tilde{f}:{\mathbb {V}}_n^{(2)}\times {\mathbb {F}}_2\rightarrow {\mathbb {Z}}_{2^k}$ given as

13 $$\begin{aligned} \tilde{f}(X,z) = a_0(X) + 2a_1(X) + \cdots + 2^{k-3}a_{k-3}(X) + z2^{k-2} + 2^{k-1}(a_{k-1}(X)+za_{k-2}(X)) \end{aligned}$$

is a gbent function. (As seen from the context, the last addition is in ${\mathbb {F}}_2$.)

#### Remark 10

Given any gbent function for $p=2$ and *n* odd, with Proposition 8 we obtain a gbent function in even dimension $n+1$. As seen in (13), the resulting gbent function is of a very special form. In general, a gbent function in even dimension $n+1$ does not correspond to a gbent function in dimension *n*, hence cannot be used for the construction of a gbent function in odd dimension.

#### Remark 11

Let *n* be odd and let $f(X) = a_0(X) + 2a_1(X) + \cdots + 2^{k-2}a_{k-2}(X) + 2^{k-1}a_{k-1}(X)$ be a gbent function from ${\mathbb {V}}_n^{(2)}$ to ${\mathbb {Z}}_{p^k}$. By Corollary 3 in [15], $a_{k-1}$ and $a_{k-1} + a_{k-2}$ are semibent functions for which the supports of their Walsh transforms are disjoint. Then $(z+1)a_{k-1}(X) + z(a_{k-1}+a_{k-2})(X) = a_{k-1}(X) + za_{k-2}(X)$ is bent, see [17].

According to Proposition 8, our strategy is to construct gbent functions $(h,\mathcal {P}_h)$ for a bent function $h:{\mathbb {V}}_{n+1}^{(2)}\rightarrow {\mathbb {F}}_2$ which

(i): has a decomposition of the form $h(X,z) = a_{k-1}(X) + za_{k-2}(X)$,
(ii): is admissible relative to a partition $\mathcal {P}_h$ of ${\mathbb {V}}_n^{(2)}\times {\mathbb {F}}_2$, which corresponds to the preimage set partition of a function $G:{\mathbb {V}}_n^{(2)}\times {\mathbb {F}}_2\rightarrow {\mathbb {F}}_2^{k-1}$ of the form $G(X,z) = (z,a_{k-3}(X),\ldots ,a_0(X))$ for some Boolean functions $a_j:{\mathbb {V}}_n^{(2)}\rightarrow {\mathbb {F}}_2$ and some integer *k*.

We use the following easily verified lemma.

#### Lemma 2

For Boolean functions $a_j:{\mathbb {V}}_{n-1}^{(2)}\rightarrow {\mathbb {F}}_2$, $j=0,\ldots ,k-3$, let $G_0:{\mathbb {V}}_{n-1}^{(2)}\rightarrow {\mathbb {F}}_2^{k-2}$, $G_1:{\mathbb {V}}_{n-1}^{(2)}\times {\mathbb {F}}_p\rightarrow {\mathbb {F}}_2^{k-2}$ and $G:{\mathbb {V}}_{n-1}^{(2)}\times {\mathbb {F}}_2\times {\mathbb {F}}_2\rightarrow {\mathbb {F}}_2^{k-1}$, be defined as

$$\begin{aligned} G_0(x)&= (a_{k-3}(x),\ldots ,a_0(x)),\quad G_1(X) = G_1(x,y) = (a_{k-3}(x),\ldots ,a_0(x)),\;\text{ and } \\ G(X,z)&= G(x,y,z) = (z,a_{k-3}(x),\ldots ,a_0(x)). \end{aligned}$$

If $G_0$ has preimage set partition $\{P_0,\ldots ,P_r\}$, $r = 2^{k-2}-1$, then

- $G_1$ has preimage set partition $\{P_0\times {\mathbb {F}}_2,\ldots ,P_r\times {\mathbb {F}}_2\}$, and
- *G* has preimage set partition $\{P_0\times {\mathbb {F}}_2\times \{0\},\ldots ,P_r\times {\mathbb {F}}_2\times \{0\}, P_0\times {\mathbb {F}}_2\times \{1\},\ldots ,P_r\times {\mathbb {F}}_2\times \{1\}\}$.

Note that some of the preimage sets in the above partitions may be empty.

Let $g_0,g_1,\ldots ,g_{p-1}$ be bent functions from ${\mathbb {V}}_s^{(p)}$ to ${\mathbb {F}}_p$, then $h:{\mathbb {V}}_s^{(p)}\times {\mathbb {F}}_p\times {\mathbb {F}}_p\rightarrow {\mathbb {F}}_p$ given as $h(x,y,z) = g_z(x)-yz$ is bent. This construction of a bent function in $s+2$ variables from *p* bent functions in *s* variables, is called the *generalized Maiorana-McFarland* construction. We refer to [13, 14]. When $p = 2$, then

14 $$\begin{aligned} h(x,y,z)&= g_z(x)+yz = g_0(x)(z+1) + (g_1(x)+y)z \nonumber \\&= \tilde{g}_0(x,y) + z(\tilde{g}_0(x,y) + \tilde{g}_1(x,y)), \end{aligned}$$

where $\tilde{g}_0(x,y) = g_0(x)$ and $\tilde{g}_1(x,y) = g_1(x)+y$. We remark that $\tilde{g}_0(x,y)$ and $\tilde{g}_1(x,y)$ are semibent functions. Observe that setting $a_{k-1}(X) = \tilde{g}_0(X)$ and $a_{k-2}(X) = \tilde{g}_0(X) + \tilde{g}_1(X)$, the Boolean generalized Maiorana-McFarland bent function *h* in (14) is decomposed as $h(X,z) = a_{k-1}(X) + za_{k-2}(X)$. Hence *h* is of the form required for constructing gbent functions $\tilde{f}$ given as in (13), and subsequently, by Proposition 8, for constructing gbent functions for $p=2$ and *n* odd.

In [19], partitions $\mathcal {P}_h$ for generalized Maiorana-McFarland bent functions *h* are investigated. As we are interested in constructing gbent functions for $p=2$ and *n* odd, we fix *n* to be an odd integer and state the required result from [19] for the case that $p=2$.

#### Lemma 3

 [19, Theorem 3.2] Let $g_0,g_1$ be bent functions from ${\mathbb {V}}_{n-1}^{(2)}$ to ${\mathbb {F}}_2$, and let $g_j$, $j = 0,1$, be admissible relative to the partition

$$\begin{aligned} \mathcal {P}_{g_j} = \{P_1^{(j)},P_2^{(j)},\ldots , P_{r_j}^{(j)}\},\; j = 0,1, \end{aligned}$$

of ${\mathbb {V}}_{n-1}^{(2)}$. Then the generalized Maiorana-McFarland bent function $h(x,y,z) = g_z(x)+yz$ from ${\mathbb {V}}_{n-1}^{(2)}\times {\mathbb {F}}_2^2$ to ${\mathbb {F}}_2$ is admissible relative to the partition $\mathcal {P}_h$ of ${\mathbb {V}}_{n-1}^{(2)}\times {\mathbb {F}}_2^2$,

$$\begin{aligned} \mathcal {P}_h&= \{P_i^{(j)}\times {\mathbb {F}}_2\times \{j\}\,:\, j = 0,1,\,1\le i\le r_j\},\;\text{ where } \\ P_i^{(j)}\times {\mathbb {F}}_2\times \{j\}&= \{(x,y,z) \in {\mathbb {V}}_{n-1}^{(2)}\times {\mathbb {F}}_2^2\,:\,x\in P_i^{(j)}, y\in {\mathbb {F}}_2, z = j\}. \end{aligned}$$

For the construction of gbent functions of the form given in (13) and subsequently of Boolean gbent functions in an odd number of variables, we will apply the following special case of Lemma 3.

#### Proposition 9

Let $g_0$, $g_1$ be bent functions from ${\mathbb {V}}_{n-1}^{(2)}$ to ${\mathbb {F}}_2$, which are admissible relative to the same partition $\mathcal {P}_{g_0} = \mathcal {P}_{g_1} = \{P_1,P_2,\ldots ,P_r\}$ of ${\mathbb {V}}_{n-1}^{(2)}$. Then the generalized Maiorana-McFarland bent function $h(x,y,z) = g_z(x)+yz$ from ${\mathbb {V}}_{n-1}^{(2)}\times {\mathbb {F}}_2^2$ to ${\mathbb {F}}_2$ is admissible relative to the partition$\mathcal {P}_h$ of ${\mathbb {V}}_{n-1}^{(2)}\times {\mathbb {F}}_2^2$,

15 $$\begin{aligned} \mathcal {P}_h =&\{ P_1\times {\mathbb {F}}_2\times \{0\},P_2\times {\mathbb {F}}_2\times \{0\},\ldots ,P_r\times {\mathbb {F}}_2\times \{0\}, \nonumber \\&P_1\times {\mathbb {F}}_2\times \{1\},P_2\times {\mathbb {F}}_2\times \{1\},\ldots , P_r\times {\mathbb {F}}_2\times \{1\} \}. \end{aligned}$$

#### Proof

The assertion follows directly from Lemma 3 with the assumption that $\mathcal {P}_{g_0} = \mathcal {P}_{g_1} = \{P_1,P_2,\ldots ,P_r\}$. $\square$

We can summarize the construction of a gbent function $\tilde{f}$ from ${\mathbb {V}}_{n+1}^{(2)}$ to ${\mathbb {Z}}_{2^k}$ of the required shape given as in (13) as follows:

We start with a gbent function $g(x)=a_0(x) + 2a_1(x) + \cdots + 2^{k-2}a_{k-2}(x)$ from ${\mathbb {V}}_{n-1}^{(2)}$ to ${\mathbb {Z}}_{p^{k-1}}$ with partition $\mathcal {P}^g_{a_{k-2}}$. We then pick two bent functions $g_0,g_1$ from the affine bent function space $\mathcal {A}(a_{k-2},\mathcal {P}^g_{a_{k-2}})$, and form the generalized Maiorana-McFarland function $h:{\mathbb {V}}_{n-1}^{(2)}\times {\mathbb {F}}_2^2\rightarrow {\mathbb {F}}_2$ given as in (14). Note that then $\mathcal {P}_{g_0} = \mathcal {P}_{g_1} = \mathcal {P}^g_{a_{k-2}}$, and $\mathcal {A}(g_0,\mathcal {P}_{g_0}) = \mathcal {A}(g_1,\mathcal {P}_{g_1}) = \mathcal {A}(a_{k-2},\mathcal {P}^g_{a_{k-2}})$. The generalized Maiorana-McFarland function $h:{\mathbb {V}}_{n-1}^{(2)}\times {\mathbb {F}}_2^2\rightarrow {\mathbb {F}}_2$ given as in (14) is then admissible relative to the partition $\mathcal {P}_h$ in (15).

We arrive at the following corollary.

#### Corollary 1

For an odd integer *n*, let $g(x) = a_0(x) + 2a_1(x) + \cdots + 2^{k-3}a_{k-3}(x) + 2^{k-2}a_{k-2}(x)$ be a gbent function from ${\mathbb {V}}_{n-1}^{(2)}$ to ${\mathbb {Z}}_{2^{k-1}}$ with partition$\mathcal {P}^g_{a_{k-2}} = \{P_0,P_1,\ldots ,P_r\}$, $r=2^{k-2}-1$, and let $g_0,g_1$ be bent functions from the affine bent function space $\mathcal {A}(a_{k-2},\mathcal {P}^g_{a_{k-2}})$.

(i): Let $h:{\mathbb {V}}_{n+1}^{(2)}\rightarrow {\mathbb {F}}_2$ be the generalized Maiorana-McFarland bent function $h(x,y,z) = g_z(x)+yz$, then *h* is admissible relative to the partition $\mathcal {P}_h$ given as in (15). The gbent function $(h,\mathcal {P}_h)$ from ${\mathbb {V}}_{n+1}^{(2)}$ to ${\mathbb {Z}}_{p^k}$ has a representation as
  $$\begin{aligned} \tilde{f}(x,y,z) = a_0(x) + 2a_1(x) + \cdots + 2^{k-3}a_{k-3}(x) + 2^{k-2}z +2^{k-1}h(x,y,z). \end{aligned}$$
(ii): The function
  $$\begin{aligned} f_1(x,y) = a_0(x) + 2a_1(x) + \cdots + 2^{k-3}a_{k-3}(x) + 2^{k-2}(g_0(x)+g_1(x)+y) + 2^{k-1}g_0(x) \end{aligned}$$
  is a gbent function from ${\mathbb {V}}_n^{(2)}$ to ${\mathbb {Z}}_{p^k}$. In particular,
  $$\begin{aligned} f_2(x,y) = a_0(x) + 2a_1(x) + \cdots + 2^{k-3}a_{k-3}(x)+2^{k-2}(a_{k-3}(x)+y) + 2^{k-1}a_{k-2}(x) \end{aligned}$$
  is a gbent function from ${\mathbb {V}}_n^{(2)}$ to ${\mathbb {Z}}_{2^k}$.

#### Proof

(i) follows readily with Proposition 9 and Lemma 2. With Proposition 8 we obtain from the gbent function $\tilde{f}:{\mathbb {V}}_{n+1}^{(2)}\rightarrow {\mathbb {Z}}_{2^k}$ in (i), which is of the form (13), the gbent function $f_1$ from ${\mathbb {V}}_n^{(2)}$ to ${\mathbb {Z}}_{2^k}$. Note that the Boolean functions $a_i$, $i = 0,\ldots ,k-3$, depend only on *x*, but one may also see them as functions in the variables *x*, *y*. The special case $f_2$ we obtain with $g_0 = a_{k-2}$ and $g_1 = a_{k-2} + a_{k-3}$, both bent functions in $\mathcal {A}(a_{k-2},\mathcal {P}^g_{a_{k-2}})$. $\square$

#### Remark 12

Whereas Proposition 8 yields a Boolean gbent function in an even number of variables from any Boolean gbent function in an odd number of variables, with Corollary 1 we obtain a Boolean gbent function in an odd number of variables from any Boolean gbent function in an even number of variables.

## Data Availability

No datasets were generated or analysed during the current study.
